# Neural Correlates of Gender Differences in Reputation Building

**DOI:** 10.1371/journal.pone.0106285

**Published:** 2014-09-02

**Authors:** Francesca Garbarini, Riccardo Boero, Federico D'Agata, Giangiacomo Bravo, Cristina Mosso, Franco Cauda, Sergio Duca, Giuliano Geminiani, Katiuscia Sacco

**Affiliations:** 1 Department of Psychology, University of Turin, Turin, Italy; 2 Los Alamos National Laboratory, Los Alamos, New Mexico, United States of America; 3 Department of Neuroscience, University of Turin, Turin, Italy; 4 CCS-fMRI, Koelliker Hospital, Turin, Italy; 5 Department of Social Studies, Linnaeus University, Växjö, Sweden; 6 Neuroscience Institute of Turin (NIT), University of Turin, Turin, Italy; University of Bologna, Italy

## Abstract

Gender differences in cooperative choices and their neural correlates were investigated in a situation where reputation represented a crucial issue. Males and females were involved in an economic exchange (trust game) where economic and reputational payoffs had to be balanced in order to increase personal welfare. At the behavioral level, females showed a stronger reaction to negative reputation judgments that led to higher cooperation than males, measured by back transfers in the game. The neuroanatomical counterpart of this gender difference was found within the reward network (engaged in producing expectations of positive results) and reputation-related brain networks, such as the self-control network (engaged in strategically resisting the temptation to defect) and the mentalizing network (engaged in thinking about how one is viewed by others), in which the dorsolateral prefrontal cortex (DLPFC) and the medial (M)PFC respectively play a crucial role. Furthermore, both DLPFC and MPFC activity correlated with the amount of back transfer, as well as with the personality dimensions assessed with the Big-Five Questionnaire (BFQ-2). Males, according to their greater DLPFC recruitment and their higher level of the BFQ-2 subscale of Dominance, were more focused on implementing a profit-maximizing strategy, pursuing this target irrespectively of others' judgments. On the contrary, females, according to their greater MPFC activity and their lower level of Dominance, were more focused on the reputation *per se* and not on the strategic component of reputation building. These findings shed light on the sexual dimorphism related to cooperative behavior and its neural correlates.

## Introduction

Theoretical research on the evolution of human cooperation highlighted the importance of how one is viewed by others, i.e., reputation [1]. Social scientists and evolutionary biologists have long investigated why humans cooperate with genetically unrelated individuals, a distinguishing feature of our species [2]. They identified reputation as one of the key mechanisms explaining human altruism through the use of indirect reciprocity strategies [3]. Converging experimental evidence suggested that humans are sensitive to the possibility of establishing a reputation [2], and that an individual's motivation to acquire a good reputation might drive cooperation through indirect reciprocity [4]. Social psychological studies showed that social approval has a profound impact on everyday decision-making [5]. Human subjects are motivated to present themselves in a positive manner or to engage in prosocial behavior when their perception of being watched by others is enhanced. For instance, during charitable donation simulations, donation rates increased with the presence of observers and neuroimaging results revealed that brain activity before the choice was significantly affected by the presence of observers [6]. Within this framework, Boero *et al*. [7] showed that reputation building significantly affects behavior in social dilemma situations and that subjects promptly react to others' judgments, especially negative ones, by modifying their previous behavior and increasing cooperative actions.

Previous neuroimaging studies showed that activities in the reward-related area, notably in the striatum, are modulated by the partner's reputation priors [8] and that the striatum is involved in processing the partner's reputation during an iterated economical interaction [9]. Also the acquisition of a good reputation robustly activates reward-related brain areas, overlapping with those activated by monetary rewards [10]. When functional Magnetic Resonance Imaging (fMRI) tasks placed a high demand on reputational processing, the “mentalizing” network, and in particular the medial prefrontal cortex (MPFC), is recruited. This area has been suggested to play a specific role in self-referential thinking [11] and specifically in the elaboration of how one is viewed by others [12]. Self-control processes have also been explored in reference with reputation building behavior. Knoch *et al*. [13] showed that virtual lesions with low-frequency repetitive transcranial magnetic stimulation (rTMS) of the right dorsolateral prefrontal cortex (DLPFC), which are involved in self-control and planning, reduced the participants' ability to build a favorable reputation. Subjects no longer seemed to be able to resist the temptation to defect, even if they knew that this may had detrimental effects on their reputation.

Although cooperation is highly developed in human beings, individual levels of cooperativeness can vary. Especially relevant are gender differences, with females showing higher cooperativeness in comparison with males across nations and cultures [14]. Because human cooperativeness has been thought to evolve with genes [2], specific effects of X-chromosome linked genes on social cognition and on brain morphology may, at least in part, explain such sex differences [15]. Psychosocial researches stressed the link between cooperation and reputation, suggesting that the social signal sent to the peers (reputation) is the driving force of gender differences in cooperative behaviors, with men who prefer to signal to their group members that they are tough, while women prefer to signal they are inclined to cooperation [16], [17]. A different line of research suggested that males tend to interact in groups of unrelated same-sex peers more than females, while females form isolated one-on-one relationships, in which social exclusion (ostracism) constitutes a typical strategy [18]. If social exclusion is utilized by females more than males, females should be more sensitive to its occurrence and hence more carefully control their own reputation.

Although the role of reputation in cooperative behavior, along with its neurological correlates, has been extensively explored, previous studies provided no evidence on gender differences in reputation building, which represents the main focus of this work. More specifically, we investigated gender differences at both the behavioral and the neuro-functional level in a situation where economic and reputational payoffs have to be balanced in order to increase personal welfare. We designed a fMRI experiment to investigate two crucial components of reputation: (a) subject reactions to judgments by others (*Reaction phase*) and (b) subject reputation building choices (*Choice phase*). We based our experiment on the trust game presented by Knoch *et al*. [13], where two players faced a social dilemma. The game was played in two experimental conditions: (i) a control, No-Reputation treatment and (ii) a Reputation treatment, offering to participants the possibility of employing reputation building strategies (see the Methods section).

Participants' personality profile was investigated using the Big Five Questionnaire (BFQ), which summarizes individual differences in enduring patterns of thoughts, feelings, motivational reactions and behaviors [20]. Being interested in gender differences within our sample, we considered those personality traits, including Energy (Dynamism and Dominance) and Friendliness (Cooperation and Social Harmony), known to show different ratings between males and females [21], [22].

The results of previous studies [7], [13], [19], [23] led us to expect that, at the behavioral level, (i) cooperation (back transfer) will significantly increase in Reputation treatments compared to the No-Reputation ones and (ii) subjects will increase their back transfer after a negative judgment while they will decrease it after a positive judgment. At the neuro-functional level, we expect that our fMRI paradigm will highlight the neural correlates of reputation building in both the choice (cooperative and no-cooperative) and the reaction (to others' judgments) phases of the task. Crucially, both at the behavioral and at the neuro-functional level, we expect that our task will be able to show how gender differences modulate the reputation building processing. In particular, if females are more sensitive to reputation than males [16]–[18], we expect that females will modify their behavior more than males when a reputation building opportunity is present. We hypothesize that the neuroanatomical counterpart of this gender difference can be found within previously described reputation-related brain networks, such as the self-control and the mentalizing network, in which the DLPFC [13] and the MPFC [12] respectively seems to play a crucial role. The reward network is a further candidate in the modulation of gender behavior, given its specific function in producing expectations of positive results [8]–[10]. More generally, our goal is to verify whether the magnitude of the reputation-related brain activity correlates with gender differences in both the cooperative behavior and the personality traits.

## Methods

### Subjects

We recruited 16 healthy subjects (8 males, mean age = 26.2, SD = 4.1; 8 females, mean age = 24.5, SD = 2.8). All subjects had no history of psychiatric or neurological illness. The protocol was approved by the local ethics committee (“Comitato di Bioetica d'Ateneo”, University of Turin, Italy), and all subjects gave their written informed consent for the study, which was performed at the Koelliker Hospital in Turin, Italy.

### The trust game

The first player (the “investor”) receives an endowment of 10 monetary units (MU) and can choose whether to transfer 1, 4, 7, or 10 MU to the second one (the “trustee”), keeping the remainder for herself. In the Choice phase the trustee receives the transferred sum multiplied by four and can choose the amount to return to the investor. To keep the game simple, the trustee's choice is limited to three options: (i) to send back nothing; (ii) to send back an amount equal to the one transferred by the investor; (iii) to send back an amount equalizing the payoff between the two players (Fig. S1A). All the possible combinations between the investor and trustee choices are summarized in Tab. S1 in File S1.

Being interested in the response to reputational opportunities, all participants in our experiment played only as trustees, while the investors were artificial agents programmed to simulate the standard behavior of people playing this game (see details in File S1). Participants under MRI scanning were induced to believe that they were interacting with real subjects through a video showing other players in an experimental room, ready to play in front of their computer monitors.

The game was played in two conditions: a No-Reputation and a Reputation treatment. In the Reputation treatment, the simulated investors expressed a judgment (“positive” or “negative”) on trustee behavior knowing their choices in the past three rounds. The judgment was transmitted to each trustee and used by the subsequent investors to modulate their choices. In this Reaction phase, the subject could see on the screen her/his own picture along whit the reputation judgment that she/he received by the investor (Fig. S1B). Consistently with the behavior observed in past studies using real subjects [7], [19], the artificial investors sent higher sums to players in good standing and awarded “positive” judgments to trustees sending back a significant share of their endowment (see details in the File S1). Participants played two instances of the game, each formed by 10 no-reputation periods plus 13 reputation periods (the reputation mechanism became active after 3 periods). They were induced to believe that their opponents changed after each exchange and that the only information that was transmitted from period to period was the judgments they received (an information actually corresponding to the programmed behavior of artificial agents).

### Experimental procedure

Subjects executed tasks within a 1.5-Tesla magnetic resonance (MR) scanner (Intera, Philips Healthcare, Best, The Netherlands) with a Philips SENSE high-field, high resolution (MRIDC) 8 channels head coil optimized for functional imaging. A head coil-mounted display system (IFIS-SA, Invivo, Gainesville, FL) was used to present visual stimulation via E-Prime software (Psychology Software Tools, Inc., Pittsburgh, PA), which also ensured synchronization with the MR scanner and the behavioral data collection. Behavioral data were collected using an MR-compatible response box: in all trials, subjects had to press one of 3 possible buttons to give their answer.

### fMRI paradigm

Each event of the fMRI paradigm started with a blank screen simulating the Investor's (A) thinking (10 sec), then the amount of monetary units (MU) invested by A was presented (4 sec). In the C*hoice phase*, the participant chose, using an fMRI compatible response box, how many MU back-transfer to the Investor (nothing; the same amount sent by A; an amount that equalize payoff between A and B) (maximum 6 sec.). After the participant's choice, different screens were shown: blank screen, simulating the results computation (10 sec.); results screen, presenting the outcome of the round (6 sec.); blank screen, simulating (only in the Reputation treatment) the Investor's decision about which evaluation award to the Trustee's (10 sec.). In the R*eaction phase* the reputation outcome was shown (in the No-Reputation treatment: “no reputation has been assigned”; in the Reputation Treatment: “good reputation” or “bad reputation”) (6 sec.).

Each trial lasted 52 seconds. We performed 2 runs per subject, each composed of 10 trials in the No-Reputation treatment followed by 13 trials in the Reputation treatment. A 14 second screen was inserted to specify whether the next game was with or without reputation. The total protocol lasted about 40 minutes.

### Training pre-fMRI

In a training period prior to fMRI, subjects viewed the task instructions on a computer monitor in the control room, and then performed the requested task. All subjects in the experiment always played as trustees. However, in the training period, to better understand the reputation mechanism, they also performed practice blocks as investors. No one reported problems learning the tasks.

### fMRI data acquisition

Data acquisition was performed using a 1.5-Tesla MR scanner (Intera, Philips Healthcare, Best, The Netherlands) with a Philips SENSE high-field, high-resolution (MRIDC) 8 channels head coil optimized for functional imaging. Functional T2*-weighted images were acquired using an echo planar imaging (EPI) sequence, with a repetition time (TR) of 2000 ms, an echo time (TE) of 60 ms, and a 90° flip angle. The acquisition matrix was 64×64; the field of view (FoV) was 256 mm square. For each run, a total of 612 “volumes” were acquired for a total duration of about 20 minutes per run. Each volume consisted of 25 axial slices, parallel to the anterior–posterior (AC-PC) commissure line and covering the whole brain; the slice thickness was 4 mm with a 0.5 mm gap. Three volumes were imaged (but not collected) at the beginning of each run to reach a steady-state magnetization, before subsequent acquisition of the experimental data.

In the same session, a set of three-dimensional high-resolution T1-weighted structural images was acquired for each participant. This data set was acquired using a Fast Field Echo (FFE) sequence, with a repetition time (TR) of 2500 ms, the shortest echo time (TE), and a 30° flip angle. The acquisition matrix was 256×256; the field of view (FoV) was 256 mm square. The data set consisted of 160 contiguous sagittal images covering the whole brain, with a voxel size of 1 mm x 1 mm x 1 mm with an acquisition time of about 6 minutes.

### fMRI data analysis

We analyzed the fMRI data using BrainVoyager QX 2.3 (Brain Innovation, Maastricht, The Netherlands) with the following preprocessing steps: mean intensity adjustment, head motion correction, slice scan time correction, spatial data smoothing (Gaussian full width at half maximum FWHM = 4 mm), linear trend removal, high pass temporal filtering (cut-off>0.004 Hz) and temporal smoothing (FWHM  = 2.8 s). After preprocessing, the fMRI data set for each subject was coregistered with their 3D high-resolution structural scan, which was transformed into Talairach space. Using the anatomical-functional coregistration matrix and the determined Talairach reference points, we transformed the preprocessed fMRI data into Talairach space. The following procedure was performed for each task condition. A single design matrix was specified for all subjects, and each defined box-car time course was convolved with a predefined hemodynamic response function (HRF) to account for the hemodynamic delay [24]. These reference time courses were entered into a General Linear Model (GLM) analysis to yield beta parameter estimates for subsequent group statistics. The regressors specified in the matrix were the Reaction phase and the Choice phase. Every regressor had a No-Reputation and a Reputation version. A more complex model, also including the two levels (Negative or Positive) of the Reaction phase as regressors, was tried doing preliminary analysis, but it was unable to better explain our data (see details in File S1). Only 10 trials of the Reputation treatment were analyzed as the first 3 among 13 did not have an assigned reputation. At the group level, a 2×2 mixed factors ANOVA with one between factor “Gender”, with two levels (Males; Females), and one within factor “Reputation”, with two levels (Reputation; No-Reputation) was performed. The ANOVA was computed within a random effects GLM framework, with a regressor per subject, to yield brain activation maps. Tests for main and interaction effects were computed at a statistical threshold of p<0.005 and then corrected for multiple comparisons (p_cor_<0.05) using cluster-size thresholding [25].

In order to verify that the gender factor had a significant effect on brain activity, irrespective of behavioral performance, we performed an ANCOVA with one between factor “Gender” (with two levels: Males; Females), one within factor “Reputation” (with two levels: Reputation, No-reputation) and a covariate variable “Behavior”. To define the “Behavior” covariate, we employed the average back transfer difference between Reputation and No-Reputation treatments. This is the main behavioral variable used to analyze reputation building, which reports a quantitative assessment of the changes in back transfers due to the effect of reputation. Since the BrainVoyager QX software, used to perform our analysis, did not support an ANCOVA model involving both between and within factors (only allowing to estimate simple models with one between factor and a covariate, see http://www.brainvoyager.com), we estimated our ANCOVA using the beta values extracted from the main results (the previously described 2×2 mixed factor ANOVA Gender x Reputation). Beta values were extracted from 5 mm spheres around peaks. As the aim was to disentangle the effect of gender from the effect of behavior, we focused on the results showing both a significant Gender effect and a significant Interaction (Gender x Reputation) effect. Hence, we performed the ANCOVA for each area shown in Table 1 under the sections “Gender factor- Males>Females” and “Interaction Gender x Reputation” and in Table 2 under the sections “Gender factor- Males>Females” and “Gender factor- Females>Males”.

**Table 1:** Choice phase: 2×2 ANOVA results.

Cluster	Brain Region	Ke	L/R%	x	y	z	t	P
**REPUTATION FACTOR - REPUTATION > NO-REPUTATION**								
1	R Precentral Gyrus (BA 4) Include: Middle Frontal Gyrus (BA 6)	8771	0/100	48	−8	48	7.40	0.000002
2	R Middle Frontal Gyrus (BA 9, 46) Include: Middle Frontal Gyrus (BA 10)	6384	0/100	23	52	18	5.91	0.000028
3	L Precuneus (BA 7) Include: PCC (BA 30,31), ACC (BA 24,32), Fusiform Gyrus (BA 37), Parahippocampal Gyrus (BA 36), Inferior Occipital Gyrus (BA 17), Middle Occipital Gyrus (BA 18, 19), Angular Gyrus (BA 39), Supramarginal Gyrus (BA 40), Postcentral Gyrus (BA 3), Precentral Gyrus (BA 4, 6), Cerebellum [IV-V, VI]	113456	55/45	−28	−59	39	11.44	<0.000001
4	R Caudate Include: Insula (BA 13), Putamen	2239	0/100	26	19	9	5.62	0.000048
5	R Thalamus Include: Globus Pallidus, Insula (BA 13), Putamen, Middle Frontal Gyrus (BA 9), Superior Frontal Gyrus (BA 10)	17081	73/27	2	−12	6	8.45	<0.000001
**GENDER FACTOR - MALES > FEMALES**								
1	R Cerebellum [Crus 1] Include: Midbrain, Pons, Thalamus, Cerebellum [Crus 2],	13370	41/59	17	−44	−33	7.39	0.000002
2	R Insula (BA 13) Include: Caudate; Inferior Frontal Gyrus (BA 47)	2047	0/100	29	22	0	6.44	0.000011
3	R Middle Frontal Gyrus (BA 6) Include: Medial Frontal Gyrus (BA 6), Precentral Gyrus (BA 6)	1993	100/0	32	−8	45	5.02	0.000151
4	R Cingulate Gyrus (BA 24) Include: ACC (BA 32)	2322	48/52	2	25	30	5.91	0.000029
5	L Caudate Include: Insula (BA 13), Putamen	1316	100/0	−13	13	3	4.41	0.000508
6	L Middle Frontal Gyrus (BA 9, 10, 46)	1163	100/0	−46	31	21	4.97	0.000167
**INTERACTION GENDER x REPUTATION**								
1	R Medial Frontal Gyrus (BA 8) Include: Superior Frontal Gyrus (BA 8)	1603	34/66	11	37	42	28.10	0.000112
2	L Precuneus (BA 7)	1459	100/0	−13	−53	45	20.10	0.000515
3	L Fusiform Gyrus (BA 37) Include: Parahippocampal Gyrus (BA 36)	4423	100/0	−31	−32	−21	59.81	0.000006
4	L Middle Frontal Gyrus (BA 9, 46)	856	100/0	−37	37	24	23.17	0.000276

Reputation factor - Reputation > No-Reputation (in the upper rows). Gender factor - Males > Females (in the middle rows). Interaction Gender*Reputation (in the lower rows).

Significantly activated clusters, p<0.05, corrected for multiple comparisons using cluster-size thresholding (after p<0.005 uncorrected). ACC  =  Anterior Cingulate Cortex, PCC  =  Posterior Cingulate Cortex. Ke  =  cluster extension in mm3. (BA)  =  Brodmann Area. L  =  Left. R  =  Right. x, y, z expressed in mm. Coordinates were reported in Talairach space. Brain regions are classified using AFNI (http://afni.nimh.nih.gov) atlas. Square brackets indicate Roman nomenclature of Schmahmann et al., 1999.

**Table 2:** Reaction phase: 2×2 ANOVA results.

Cluster	Brain Region	Ke	L/R%	x	y	z	t	P
**REPUTATION FACTOR - REPUTATION > NO-REPUTATION**								
1	R Angular Gyrus (BA 40) Include: Superior Temporal Gyrus (BA 22), Supramarginal Gyrus (BA 39)	2555	0/100	41	−62	36	6.49	0.000010
2	R Medial Frontal Gyrus (BA 8) Include: Cingulate Gyrus (BA 32), Superior Frontal Gyrus (BA 8)	9067	43/57	2	34	42	6.62	0.000008
3	L Globus Pallidus	1583	94/6	−10	−2	3	5.72	0.000041
4	L Middle Frontal Gyrus (BA 10) Include: Inferior Frontal Gyrus (BA 47), Superior Frontal Gyrus (BA 10)	7629	100/0	−31	55	12	7.02	0.000004
5	L Supramarginal Gyrus (BA 39) Include: Superior Temporal Gyrus (BA 22), Angular Gyrus (BA 40), Precuneus (BA 7)	6707	100/0	−46	−56	33	8.53	<0.000001
6	L Middle Frontal Gyrus (BA 6) Include: Precentral Gyrus (BA 6)	1693	100/0	−43	4	39	5.26	0.000097
7	L Inferior Frontal Gyrus (BA 45)	1796	100/0	−46	16	15	49.7	0.000167
**REPUTATION FACTOR - NO-REPUTATION > REPUTATION**								
1	R Lingual Gyrus (BA 18) Include: Fusiform Gyrus (BA 37), Inferior Temporal Gyrus (BA 37), Parahippocampal Gyrus (BA 36), Middle Occipital Gyrus (BA 19)	11019	0/100	11	−56	3	5.81	0.000034
2	R Insula (BA 13) Include: Putamen	1615	0/100	35	−8	−6	5.59	0.000051
3	L Lingual Gyrus (BA 18), Include: Fusiform Gyrus (BA 37), Parahippocampal Gyrus (BA 36)		100/0	−46	−78	0	−521	0.000106
**GENDER FACTOR - MALES > FEMALES**								
1	L Inferior Frontal Gyrus (BA 47)	3139	100/0	−49	22	−15	4.71	0.000277
GENDER FACTOR - FEMALES > MALES								
1	R Fusiform Gyrus (BA 37) Include: Lingual Gyrus (BA18)	8003	0/100	35	−53	−12	−6.09	0.000021
2	L Superior Parietal Lobule (BA 7) Include: Precuneus (BA 7)	1441	100/0	−28	−59	45	−4.91	0.000187

Reputation factor - Reputation vs. No-Reputation (in the upper rows). Gender factor - Males vs. Females (in the lower rows).

Significantly activated clusters, p<0.05, corrected for multiple comparisons using cluster-size thresholding (after p<0.005 uncorrected). Ke  =  cluster extension in mm^3^. (BA)  =  Brodmann Area. L  =  Left. R  =  Right. x, y, z expressed in mm. Coordinates were reported in Talairach space. Brain regions are classified using AFNI (http://afni.nimh.nih.gov) atlas. Square brackets indicate Roman nomenclature of Schmahmann et al., 1999.

Moreover, we performed a path analysis through Structural Equation Modeling (SEM; see details in File S1) in order to investigate whether gender had a significant direct impact on behavior, or whether it could predict behavior only by mediation of brain areas.

Finally, we estimated the correlations between the beta values extracted from the interaction contrast (Gender x Reputation) of the Choice phase and both the behavioral (as defined for the Behavior covariate) and the personality trait results. We extracted beta value from three areas showing an interaction effect in the Choice phase, namely L DLPFC; MPFC; PCN. For each area, the average beta values difference in the two treatments (Reputation minus No-Reputation) was correlated with the average back transfer difference (Reputation and No-Reputation) and with the Energy scores. We calculated the following 10 correlations: L DLPFC – MPFC; L DLPFC – PCN; L DLPFC – ENERGY; L DLPFC – BACK TRANSFER; MPFC – PCN; MPFC – ENERGY; MPFC – BACK TRANSFER; PCN – ENERGY; PCN – BACK TRANSFER; ENERGY – BACK TRANSFER. Only 6 out of 10 survived after correction for multiple comparisons (Step-Down Finner). In particular, all four correlations involving PCN did not survived after correction. For the brain areas showing significant correlations, namely the DLPFC and the DMPFC, we also performed an ANCOVA analysis, in which the gender effect was controlled, using their beta value difference to predict back transfer. In a similar ANCOVA model, we used the Energy scores to predict both back transfers and brain activity.

To test the robustness of our interaction (Gender x Reputation) results, we repeated the GLM analysis at the individual-subject level [26]. We used the same design matrix with a more liberal uncorrected threshold of p<0.05, and we then created separate probability maps of the contrast Reputation > No reputation in the Choice phase for male and female subgroups.

### Meta-analyses

In order to have stronger bases to discuss the present results in terms of networks, we performed three meta-analyses on the main networks discussed in the paper (namely, reward, self-control and metalizing networks). We generated them using the BrainMap database, the Sleuth 2.0.3 and GingerAle 2.1 software (see methodological details in File S1). We extracted from the BrainMap database all the studies involving normal subjects that reported an activation in three Experimental Paradigm Classes of interest described below:

1. Reward Task: a behavioral experimental paradigm in which, in at least one of the conditions, subjects performed a task in which correct performance was associated with reward, often monetary reward;
2. Delay Discounting Task: a behavioral experimental paradigm that measured subject self-control, i.e., the capacity to resist the temptation of an immediately delivered small reward to obtain a larger reward delivered at variable delays;
3. Theory of Mind Task: a behavioral experimental paradigm in which the subjects had to (i) attribute mental states - beliefs, intents, desires, pretending, knowledge, etc. - to themselves and to others and (ii) to understand others' beliefs, desires and intentions as different from their own.

Meta-analyses results are shown in Fig. S2 and Tab. S2 in File S1. The list of references included in each meta-analysis is shown in File S1.

## Results

### Personality traits results

A total of 16 individuals, divided equally by gender, participated in the experiment. We assessed their personality traits on two dimensions: Energy, defined by the facets Dominance and Dynamism, and Friendliness or Agreeableness, defined through Cooperation and Social Harmony. We used the Italian version of the BFQ2 (Barbaranelli & Caprara, 2000). Participants rated the extent to which each item applied to them on a 5-point-scale (1 =  strongly disagree, 5 =  strongly agree). We found significant gender differences on the Energy trait, with males showing an average of 4.83±0.18 and females of 3.94±0.22 (Wilcoxon rank sum test: *W* = 58, *p* = 0.002 one tailed). Especially relevant were differences in the Dominance sub-trait, where men again scored significantly higher than women (4.52±0.20 vs. 3.51±0.22; *W* = 56.5, *p* = 0.006 one tailed). No significant gender difference was instead found in Friendliness.

### Behavioral results

Consistently with past research [7], [13], [19], [23], back transfers were significantly higher in the Reputation treatment than in the No-Reputation one (7.84±0.38 MU vs. 3.17±0.33 MU; Wilcoxon signed rank test on individual averages: *V* = 136, *p*<0.001 one tailed). The modal choice was to transfer back nothing in the no-reputation periods, while it became to transfer back the amount invested in the reputation ones. Note also that subjects chose to equalize payoffs more than 20% of the times in the reputation periods against 4% in the no reputation ones. See supplemental behavioral data analysis in File S1 (see also Fig. S3 and Tab. S3 in File S1).

**Figure 1:**
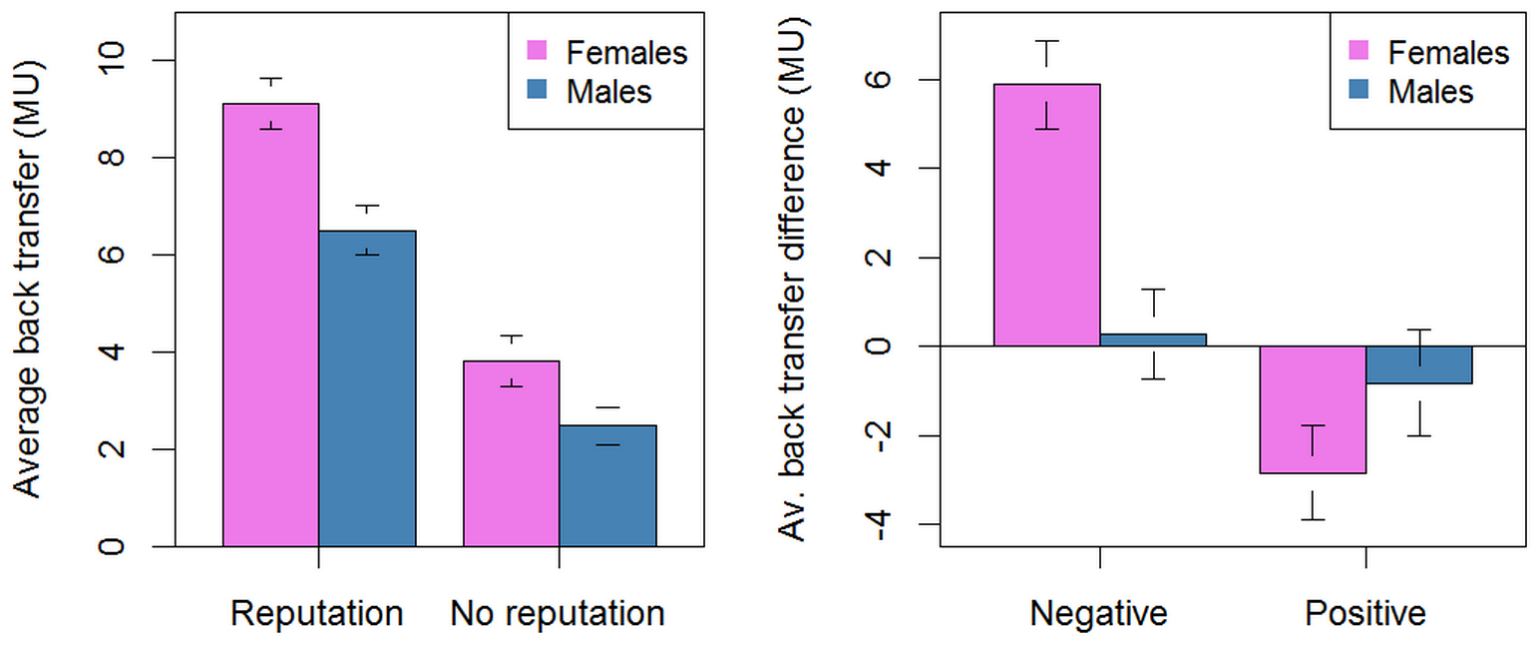
Behavioral results. The (A) panel shows the average back transfer by gender and condition, the (B) panel the average difference in back transfer following a positive/negative evaluation by gender.

Overall, females returned slightly more than males (Fig. 1A). While in the No-Reputation treatment the difference was not significant (3.82±0.53 MU vs. 2.49±0.38 MU, *W* = 37, *p* = 0.323 one tailed) it approached significance in the Reputation treatment (9.12±0.54 MU vs. 6.51±0.51 MU, *W* = 48, *p* = 0.052 one tailed).

Most of the increase in cooperation in the Reputation treatment depended on the rise in back transfer that followed negative evaluations. Here gender differences were highly significant, with females modifying their behavior more than males after both a negative and a positive evaluation (Fig. 1B). More specifically, after a negative evaluation females increased their average back transfers by 5.88±1.00 MU, males only by 0.27±1.01 MU (*W* = 52, *p* = 0.019 one tailed). After a positive evaluation, females decreased their back transfers by 2.84±1.05 MU, males by 0.82±1.19 MU (*W* = 14.5, *p* = 0.037 one tailed). Note also that no significant differences between genders were recorded in the no reputation periods, when back transfers tended to decrease over time for both groups, and in the early periods of the Reputation treatment (when no judgement was expressed), when back transfer tended to increase for all participants (gender differences: *W* = 29.5, *p* = 0.417 one tailed, and *W* = 22, *p* = 0.159 one tailed, respectively).

### fMRI results

#### Choice phase

**Figure 2:**
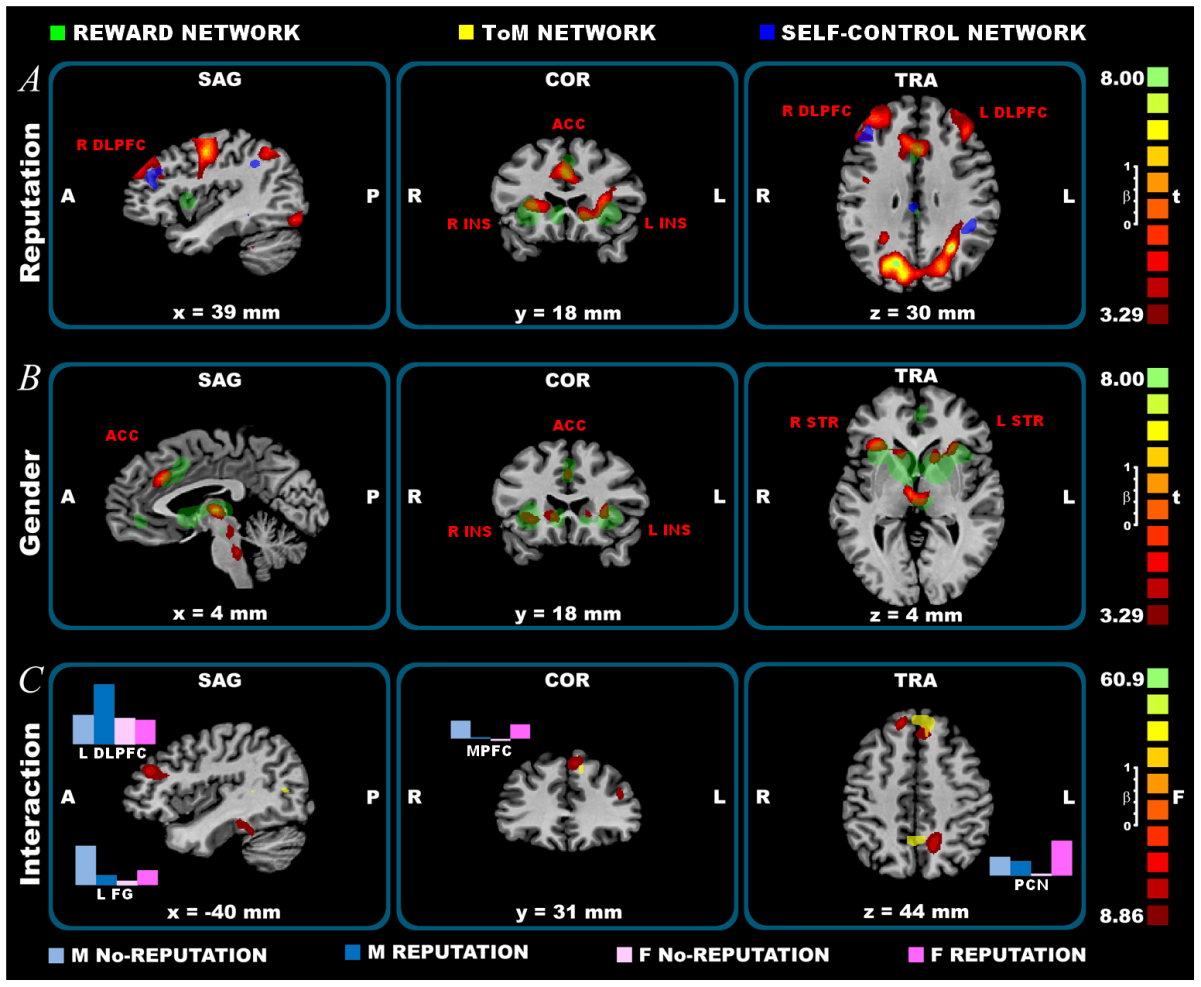
Choice phase fMRI results. From top to bottom: A) reputation effects; B) gender effects (Males>Females); C) interaction effects. Significantly activated clusters, corrected for multiple comparisons at p_cor_<0.05 using cluster-size thresholding (after p<0.005 uncorrected). A = Anterior, P =  Posterior. SAG =  Sagittal, COR =  coronal, TRA =  Transverse. L =  Left, R =  Right. ACC =  Anterior Cingulate Cortex. DLPFC =  Dorso-Lateral Pre-Frontal Cortex. FG =  Fusiform Gyrus. INS =  Insula. MPFC =  Medial Pre-Frontal Cortex. PCN =  Precuneus. STR =  Sriatum. Statistical maps overlaid on a Talairach space template. In transparent colors, the meta-analyses results are plotted: in grey the reword network; in blue the self-control network; in yellow the Theory of Mind network (ToM). In the interaction effect C), for each condition, the average beta values are plotted in blue for Males and in pink for Females.

In the Choice phase, the ANOVA results found significant effects of both the within factor “Reputation” and the between factor “Gender” and also of the Interaction “Gender x Reputation”. A greater brain activity for the Reputation treatment compared with the No-Reputation one was found in bilateral clusters, including rostral prefrontal cortex (PFC), dorsolateral (DL)PFC, anterior and posterior cingulate cortex (ACC; PCC), premotor and primary motor cortex (PMC; M1), primary somatosensory cortex (S1), inferior and superior parietal lobe (IPL; SPL), striate (V1) and extrastriate visual cortex (V2-5), the fusiform gyrus and parahippocampal gyrus, insula, cerebellum, thalamic nuclei and basal ganglia, including striatum (caudate nucleus and putamen) and globus pallidus. See Fig. 2A and Tab. 1. Our results did not show any significantly greater activity in the No-Reputation treatment compared to the Reputation treatment.

Focusing on the Gender factor, a greater brain activity for males compared with females was found in left DLPFC and in bilateral clusters, including rostral PFC, ventral lateral PFC, PMC, ACC, insula, striatum (caudate nucleus and putamen), posterior cerebellum, pons, midbrain, including ventral tegmental area (VTA) and thalamus. See Fig. 2B and Tab. 1. Our results did not show any significantly greater activity in females compared to males.

Interacting the Gender and Reputation factors, we found significant activity in the left DLPFC (BA 9, 46), the bilateral medial (M)PFC, the left precuneus, the left fusiform gyrus and bilateral parahippocampal gyrus. At post hoc comparison, we found that, only in the Reputation treatment, the left DLPFC is more active in males than females. On the contrary, in males the activity of the MPFC, precuneus, fusiform gyrus and parahippocampal gyrus was greater in the No-Reputation treatment than in the Reputation one, while females showed the opposite effect (i.e., greater activity in the Reputation treatment than in the No-Reputation one). See Fig. 2C and Tab. 1. The robustness of these interaction effects was also confirmed by single subject analyses in the contrast Reputation > No-Reputation treatment, showing the specificity of the males' recruitment of the left DLPFC (100% of males; 30% of females) and of the females' recruitment of mesial areas, such as the MPFC (0% of males; 54% of females), precuneus (13% of males; 88% of females), and occipitotemporal areas such as the fusiform gyrus (13% of males; 63% of females). See Fig. S4 and Tab. S4 in File S1.

The ANOVA results were confirmed by the ANCOVA model, carried out to verify that the gender factor had a significant effect on brain activity, irrespective of behavioral performance. Despite the introduction of the “Behavior” covariate, the effect of Gender and that of the Interaction (Gender x Reputation) remained significant in all the areas identified in ANOVA analyses. The effect of the “Behavior” covariate was significant in itself only in three areas. Moreover, in all cases the effect of Gender was greater than that of Behavior: in the Insula (partial eta squared: Behavior = 0.43; Gender = 0.7) and in the Caudate Nucleus (partial eta squared: Behavior = 0.53; Gender  = 0.84), both showing a Gender effect, and in the Fusiform Gyrus (partial eta squared: Behavior  = 0.28; Gender  = 0.44) showing an Interaction effect (see Tab. S5 in File S1). These results strongly suggest that the effect of gender in predicting brain activity remains significant over and above the effect of behavior. Furthermore, the SEM model results (see File S1) showed that gender does not have a significant direct effect on behavior. Nevertheless, gender does have a significant indirect effect on behavior, that is to say that gender predicts behavior only if mediated by brain areas.

#### Reaction phase

**Figure 3:**
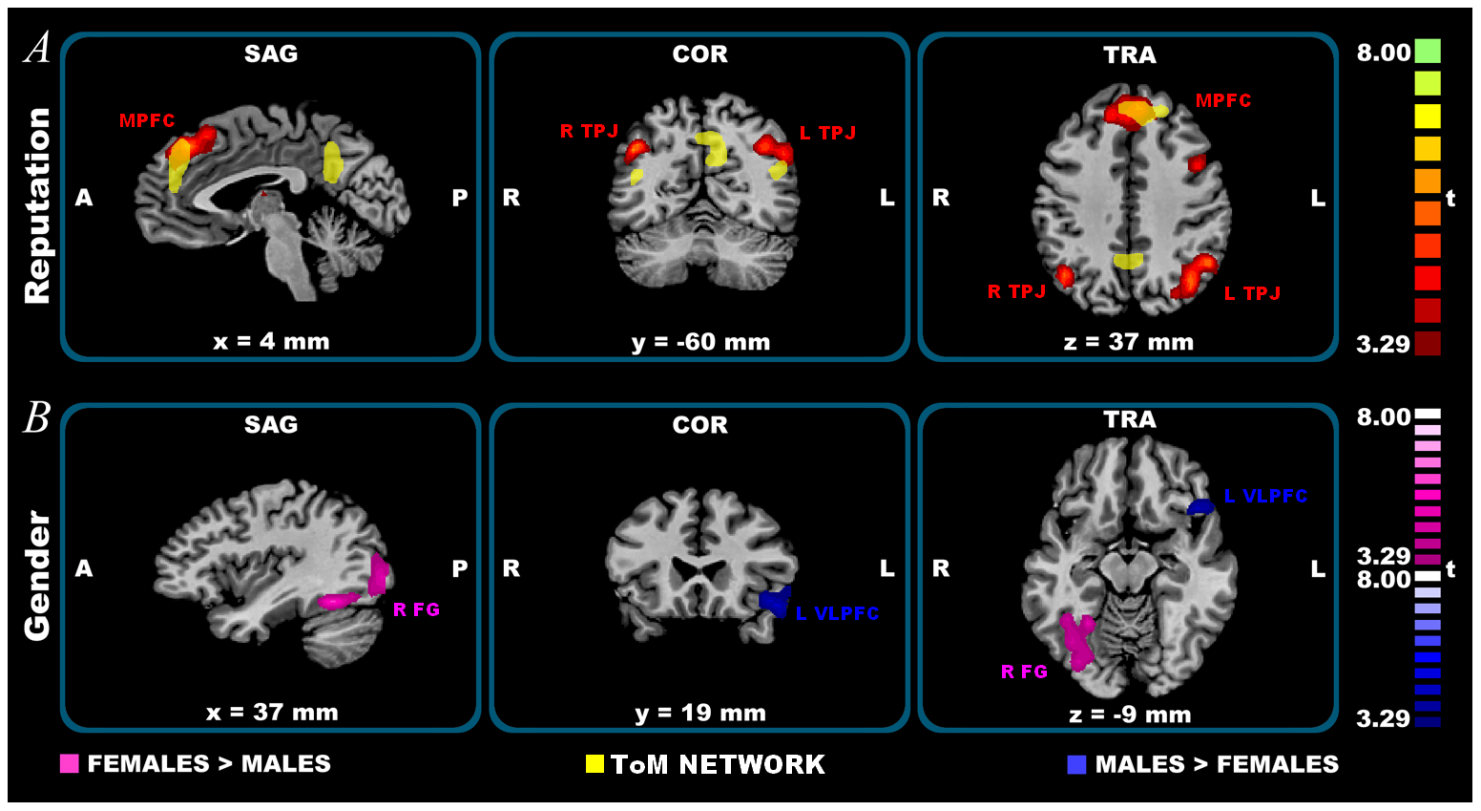
Reaction phase fMRI results. From top to bottom: A) reputation effects; B) gender effects (Males > Females in blue; Females > Males in pink). Significantly activated clusters, corrected for multiple comparisons at p_cor_<0.05 using cluster-size thresholding (after p<0.005 uncorrected). A  = Anterior, P  =  Posterior. SAG  =  Sagittal, COR  =  coronal, TRA  =  Transverse. L  =  Left, R  =  Right. FG  =  Fusiform Gyrus. MPFC  =  Medial Pre-Frontal Cortex. TPJ  =  Temporo Parietal Junction. VLPFC  =  Ventro Lateral Pre-Frontal Cortex. Statistical maps overlaid on a Talairach space template. In yellow the Theory of Mind network (ToM) is plotted.

In the Reaction phase, the ANOVA found significant effects of both the within factor “Reputation” and the between factor “Gender”; no significant effects of the Interaction “Gender x Reputation” were found. A greater brain activity for the Reputation treatment compared with the No-Reputation treatment was found in bilateral clusters, including IPL, SPL (in particular the precuneus), the superior temporal gyrus at the temporo-parietal junction (TPJ), MPFC, ACC and in left clusters, including rostral PFC and ventral lateral PFC. A greater brain activity for the No-Reputation treatment compared with the Reputation one was found in bilateral lingual gyrus, fusiform gyrus and parahippocampal gyrus. See Fig. 3A and Tab. 2.

In measuring the effect of the Gender factor, males exhibited greater brain activity than females in the left ventral lateral PFC. On the contrary, a greater brain activity for females compared with males was found in the right lingual and fusiform gyrus and in the left precuneus. See Fig. 3B and Tab. 2.

Also in the Reaction phase, the ANOVA results were confirmed by ANCOVA estimations, which showed that the Gender effects remained significant after the introduction of the “Behavior” covariate. The effect of the “Behavior” covariate was significant in itself only in one area showing a Gender effect, the SPL, where the Gender effect was nonetheless greater (partial eta squared: Behavior  =  0.5; Gender = 0.77). See details in Tab. S6 in File S1.

### Correlations between brain activity, personality traits and behavioral results

**Figure 4:**
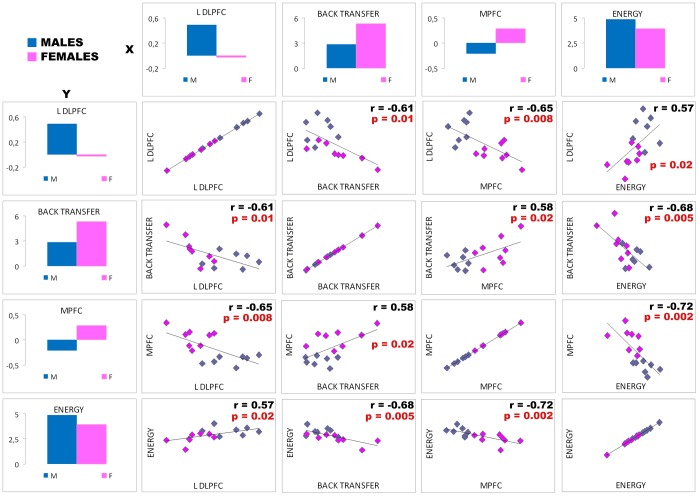
Correlations between the brain activity and behavioral and personality traits results For each subject, the average beta values difference in the two treatments (Reputation minus No-Reputation), for both the left DLPFC and the MPFC, was correlated with the average back transfer difference (Reputation and No-Reputation) and with the Energy scores. For each correlation Pearson's r and p value are reported.

Significant correlations were found between the brain activity of the PFC, in particular of the left DLPFC and the bilateral MPFC (inversely correlated each other; *r* = −0.65, *p* = 0.008) showing an interaction effect in the Choice phase, and both the Energy trait scores and the amount of back transfers (inversely correlated each other; *r* = −0.68, *p* = 0.005). The higher is the left DLPFC activity, the higher is the Energy score (*r* = 0.57, *p* = 0.02) and the lower is back transfer (*r* = −0.61, *p* = 0.01). On the contrary, the higher the MPFC activity, the higher the back transfer (*r* = 0.58, *p* = 0.02) and the lower the Energy score (*r* = −0.72, *p* = 0.002). See Fig. 4. We found that the magnitude of activity in these brain regions predicted the observed behavior in an ANCOVA model, in which the gender effect was controlled for. Both the DLPFC and the MPFC activity showed a significant effect (*p* = 0.003; *p* = 0.02, respectively) in predicting back transfer in our subjects. In a similar ANCOVA model, we also verified that the Energy scores predicted both back transfers (*p* = 0.006) and brain activity (DLPFC *p* = 0.01; MPFC *p* = 0.0003).

## Discussion

Our experiment showed that cooperation significantly increased in the Reputation treatment compared to the No-Reputation one, a result that is fully consistent with past research [7], [13], [19], [23]. Crucially, our behavioral results showed significant gender differences in reaction to reputation judgments. Both sexes showed similar levels of cooperation in the No-Reputation treatment, while the stronger reaction of females to negative judgments led them to increase back transfers more than males in the Reputation treatment. These differences in cooperative behavior likely reflect the activity of different neural circuits between genders. In particular, gender differences were found within the reward network (engaged in producing expectations of positive results), the self-control network (engaged in strategically resisting the temptation to defect) and the mentalizing network (engaged in thinking about how one is viewed by others).

We will discuss our results in terms of these networks [27], [28] and we will focus on their role in two crucial components of reputation building: the reaction to reputation judgments (Reaction phase), which in turn lead to the choice of how to manage back transfers (Choice phase). In the Reaction phase, a bilateral mentalizing network (mostly involving MPFC and TPJ) showed a significant reputation effect (Reputation > No-reputation). In the Choice phase, a bilateral reward network (mostly involving the ACC, the striatum, the insula and the VTA) showed both a reputation effect and a gender effect (males > females). Also the self-control network (mostly involving a bilateral DLPFC) showed a significant reputation effect in the Choice phase, as well as an interaction effect (Gender x Reputation) where the activity of the left DLPFC in the Reputation treatment was greater in males then in females. An interaction effect (Gender x Reputation) was also found in the mentalizing network, where the activity of the bilateral MPFC and left precuneus was greater in females then in males.

### Reaction phase

One of the most consistent discoveries in cognitive neuroscience is that the ability to “mentalize” draws on a discrete network of brain regions, comprising the MPFC, posterior aspects of the superior temporal sulcus at the TPJ level and the precuneus (see meta-analysis results in Fig. S2 a; Tab. S2 a in File S1; for a different ALE meta-analysis on mentalizing network see also [29]). This network is engaged by numerous tasks that involve mentalizing, including the so-called “Theory of Mind” (ToM), that is the reasoning about others' mental states [30]. If the elaboration of one's own reputation is indeed supported by an appreciation of others' mental states, then these regions should play an important role in our task. This is exactly what we found in the Reaction phase, where a mentalizing network, mostly involving MPFC and TPJ showed a significant reputation effect (see Fig. 3A and Tab. 2). Indeed, the high demand on reputational processing induced a recruitment of the mentalizing network, supporting the role of the MPFC in self-referential thinking [11] and specifically in the elaboration of how one is viewed by others [12]. Crucially, one component of this mentalizing network - namely, the left precuneus - showed a significant gender effect, with a greater activity in females than in males (see Fig. 3B and Tab. 2), as well as an interaction effect in the Choice phase (see below). This would suggest that, during the Reputation treatment, females were more engaged in reasoning about others' mental states and more interested in the reputation judgments expressed by the other players.

In the Reaction phase, a reputation effect was also found in the left rostral PFC (BA 10) and in the ventral lateral PFC (BA 47). The activity of these regions, and in particular of the left BA 47, has been described in the social norm violation context as modulating the current strategy to prevent the individual from engaging in inappropriate behaviors [31]. When representing the violation of a social norm, individuals form expectations of others' social disapproval, which reduce the temptation of inappropriate response options in favor of more appropriate ones. In our Reputation treatment, the representation of the investor's disapproval in the left BA 47 may have prevented the trustee's temptation to defect. This preventive mechanism also showed a gender effect: it was greater in males than in females (see Fig. 3B and Tab. 2), suggesting that the social norm violation, as other risk-taking options, is more embedded in male behavior [32] and that, in order to prevent inappropriate behaviors, males would make a greater effort than females. On the contrary, the activity of both the lingual and the fusiform gyrus, known to be implicated in the visual elaboration of the stimuli and in particular in the face identity recognition [33], [34], was greater in females (see Fig. 3B and Tab. 2), suggesting that they felt more involved when their picture was presented and their self-identity was recognized.

### Choice phase

In the Choice phase, different brain networks distinguished the Reputation treatment from the No-Reputation one. First of all, an attention network, involving bilateral visual (V1; V2–5) and parietal areas (IPL; SPL), and a hand-related motor network, mostly involving bilateral PMC, M1, S1 and cerebellum (Fig. 2A and Tab. 1) are more active in the Reputation treatment. This suggests a greater salience of the Reputation treatment in comparison with the No-Reputation one. Interestingly, two other brain networks, namely the self-control network (involving the DLPFC and the rostral PFC) and the reward network (involving the ACC, the striatum and the insula) showed significant reputation effects.

The self-control network is believed to influence decision-making by exerting an inhibitory influence on emotionally charged, impulsive and immediately rewarding choice options. Within this network, the crucial role of the DLPFC (BA 9; 46) has been described (see meta-analysis results in Fig. S2b; Tab. S2b in File S1). According to Harris and colleagues [35], the DLPFC affects self-control through two different mechanisms: attention filtering and value modulation. The functional activity of this brain region in different versions of the “delay discounting task” demonstrated its function in resisting the temptation of a smaller immediate reward *in lieu* of receiving a larger reward at a later time (e.g., for discounting of monetary gains see [36]). According to Knoch and colleagues [13], because costly reputation formation requires an override of immediate benefits, the role of DLPFC seems to be crucial in reputation building processing. The authors found that TMS disruption of the right DLPFC functionally weaken self-control capacity and thus lead to lower back-transfers also in the Reputation treatment. It is interesting to note that, in our results, the right DLPFC was commonly activated in both sexes, while the activity of the left DLPFC showed a gender effect, being greater in males than in females (see Fig. 2B and Tab. 1). The left DLPFC also showed an interaction effect, suggesting its crucial role in males' reputation behavior (see below). Within this self-control network, the interaction between the DLPFC and the orbitofrontal cortex has been described [37]. Besides the DLPFC, the activity of the rostral PFC (BA 10) also showed a significant reputation effect in our study. The role of this brain region in complex decision-making and task switching has been extensively described [38], along with its specific function in protecting the execution of long-term mental plans from immediate environmental demands and in generating new, possibly more rewarding behavioral or cognitive sequences [39].

A strong reputation effect was also found in a bilateral reward network, mostly involving ACC, striatum, insula and VTA (see meta-analysis results in Fig. S2c; Tab. S2c in File S1; for a different meta-analysis on the reword network see also [40]). These areas have been described in reward-based decision-making as involved in the expectation of a positive result, such as monetary and reputational outcomes in our task [8]–[10]. The activity of this network is clearly related to the higher expectation, characterizing the Reputation treatment, in which subjects interpreted the game outcome as a consequence of their strategic choices. The joined activity of the self-control network and the reward network in the Reputation treatment suggests that these brain regions work together to plan high future rewards.

It is interesting to note that the activation of all the reward-related areas (ACC, striatum, insula, VTA) showed a gender effect in our data, being greater in males than in females regardless of the reputational context (see Fig. 2B and Tab. 1). A previous study showed similar gender differences in reward-related decision processing under stressful conditions, and the competitive context of the trust game here proposed can be considered as such [32]. Consistently with our results, Lighthall and colleagues showed that, under stress, the activation in the dorsal striatum and in the anterior insula was increased in males relative to females. This functional difference in brain activity was thought to mirror behavioral sex differences, showing that under stress males tend to increase risk taking in pursuit of greater reward, whereas stress effects were opposite for females [32].

Crucially, in the Choice phase, we found a significant interaction effect, suggesting specific gender differences in the reputational context (see Fig. 2C and Tab. 1). In particular, when the reputation opportunity is present, males activated a component of the self-control network involved in the strategic planning, the left DLPFC, more than females, while females activated more some components of the TOM network, namely the bilateral MPFC and the left precuneus. Also, the activity of the left fusiform and parahippocampal gyrus showed the same reputation effect, but only in females. In the Reaction phase, a similar network related to the self-identity recognition showed a greater effect in females than in males. Here, in the Choice phase, female fusiform and parahippocampal gyrus activity can be attached to the memory of the self-identity, which becomes more salient when good or bad reputation is associated. Indeed the fusiform gyrus activity is known to vary with face working memory demands and the parahippocampal gyrus, considered the complement of the fusiform face area, is known to play an important role in memory encoding and retrieval activity [34]. This brain activity, together with the MPFC known to play a crucial role in self-referential processing [10], suggests that, during the Reputation treatment, female decision-making was strongly driven by thinking about how one is viewed by others. On the contrary, all these areas (MPFC, precuneus, fusiform and parahippocampal gyrus) were inhibited in the Reputation treatment with respect to the No-Reputation one in males. More specifically, the mesial inhibition of male PFC can be related to the (left) lateral activation, as suggested by the fact that the left DLPFC and the MPFC showed a reverse correlation. Crucially, both the DLPFC and the MPFC activity correlate with the amount of back transfer, as well as with the trait of Energy, and in particular with the Dominance dimension, both showing significant gender differences. In particular, the higher is the left DLPFC activity, the higher is the Energy score and the lower is the attitude to cooperate. This combination corresponds to the male profile, characterized by a bilateral recruitment of the self-control network: the stronger the activity of the additional left component of this network, the stronger seems to be the effort the subject has to make in order to resist the temptation to defect. On the contrary, the higher the MPFC activity, the higher are back transfers and the lower is the Energy score: a combination instead representing the female profile (see Fig. 4). According to the literature, Dominance is a male specific personality trait and it is related to the ability to impose the individual's point of view, regardless of others' opinions [22]. In our task, males, in line with their greater DLPFC recruitment, are more focused on implementing a strategy to maximize their profit: it seems that males take into account the other investors' judgments only as far as such opinions can affect their profit. In females, on the contrary, a lower level of Dominance corresponds to their greater sensibility of others' points of view. In our task, this translates into a higher behavioral flexibility and a stronger reaction towards the investors' judgments. This suggests that females, according to their greater MPFC activity, are focused on the reputation *per se* and not on the strategic component of the reputation building, something that indirectly leads to a more cooperative behavior. Future experiments may further check the strategic vs. not-strategic aspects of reputation and their links with gender differences by implementing under fMRI a protocol similar to the one used in Boero and colleagues [7].

## Limitations of the study

The main limitation of the present study is clearly given by its small sample size (16 subjects: 8 males and 8 females). Furthermore, given the small sample, some uncontrolled variables, as, for instance, the differences in IQ/cognitive abilities, can potentially confound the results. Nevertheless, single-subject analysis confirmed the results of group analysis, i.e., the interaction effect in the Choice phase, showing gender differences in brain activity related to reputation behavior (see Fig. S4 and Tab. S4 in File S1).

A second limitation of the study is that the order of Reputation and No-Reputation treatment was not counterbalanced. When designing the experiment, we based this decision on our previous experience with trust games, which suggests that starting with a reputation condition and then dropping the reputation opportunity leads to very low cooperation levels in the subsequent trials. In the present experiment, at a behavioral level, the sequence effect was limited in the No-Reputation treatments and absent in the Reputation treatments, leading to similar outcomes in both runs (see Fig. S3). As far as neuroimaging results are concerned, although the Reputation treatment always followed the No-Reputation one in both runs, a greater brain activity was found in the contrast Reputation > No-Reputation, in both the Choice and the Reaction phase (see Tab. 1 and Tab. 2; Fig. 2A and Fig. 3A).

## Conclusion

From an evolutionary perspective, the findings of the present study can be related to different strategies for the reproductive success, where males tend to increase risk taking in pursuit of greater opportunities, whereas females tend, on the opposite, to consolidate their reproductive opportunity avoiding risk. According to Wilson and Daly [41] some factors, such as social status and access to sexual partners, can limit men's – but not women's – reproductive success. For this reason, men would engage in same-sex aggressive behaviors. Indeed, although aggressions are death threating, men who fail to attain a sexual partner meet reproductive death [42]. On the other hand, risk-avoidance is an adaptive trait for females, serving the function of protecting physical integrity. As infants are more dependent on mothers than on fathers, a stronger selection pressure of avoiding physical injury would weigh on women [43]. In our task, larger back transfers protect players from risks, guaranteeing good reputation and, as a consequence, the reception of high investments in the future. These results may also fit a sociocultural perspective, where men and women are expected to regulate their behavior according to gender stereotypes. In particular, women are expected to be more oriented toward interpersonal relationships and therefore more prone to cooperate, whereas greater defection is expected from man for whom an egocentric orientation, motivated by personal greed, is socially tolerated [16], [17]. The understanding of the neural basis of reputation building can consolidate both these perspectives, shading light on the sexual dimorphism related to cooperative behavior.

## Supporting Information

Figure S1 **a) Trustee's decision screen (choice phase). b) Trustee's reputation outcome screen (reaction phase).** In the reputation treatment, the subject could see along his picture the reputation judgment, either “positive reputation” or “negative reputation”, that he received by the investor. In the no-reputation treatment, under the subject's picture there was the writing: “no reputation has been assigned”. The person in the picture has given written informed consent, as outlined in the PLOS consent form, to publication of his photograph. We also specified that the image used in this figure is not the original image used in the study, but a similar one used for illustrative purposes only.
(TIFF)

Figure S2 **fMRI and PET meta-analysis results.** From top to bottom: A) Theory of Mind (TOM) ALE; B) Delay Discounting Task (DDT) ALE; C) Reward ALE. Consistent ALE clusters, using p<0.01 FDR corrected for multiple comparisons, Ke>200 mm3. A =  Anterior, P =  Posterior, S =  Superior, L =  Left, R =  Right (neurological convention). Statistical maps overlaid onto a Talairach space template. From top to bottom: z = −1 mm, y = 9 mm, x = 4 mm; z = 28 mm, y = 34 mm, x = 41 mm; z = 13 mm, y = −56 mm, x = 0 mm.
(TIF)

Figure S3 **Back transfer dynamics in the game.** Vertical dashed lines mark condition changes.
(TIFF)

Figure S4 **Probability maps of the Males and Females activity in the Reputation > No-Reputation contrast in the Choice phase.** From top to bottom: males (in blue) and females (in pink) probability maps of significantly activated clusters, p<0.05 uncorrected. Statistical maps overlaid on a Talairach space template. Radiological convention: right on the left side of the figure. DLPFC  =  Dorso-Lateral Pre-Frontal Cortex. MPFC  =  Medial Pre-Frontal Cortex. PCN  =  Precuneus.
(TIF)

File S1 **Supplemental Methods and Results.** Including: Simulated agents; Matlab script; Preliminary fMRI analysis and results; Path Analysis with Structural Equation Modeling; fMRI and PET Meta-analysis; Supplemental analysis of behavioral data; Supplemental References; List of the references included in the three meta-analyses; Tables S1–S6.
(PDF)
