# Supplementary figures and images for: Neural Correlates of Gender Differences in Reputation Building
Source: PLoS One. 2014 Sep 2;9(9):e106285. doi: 10.1371/journal.pone.0106285 (PMC4152267; doi:10.1371/journal.pone.0106285)

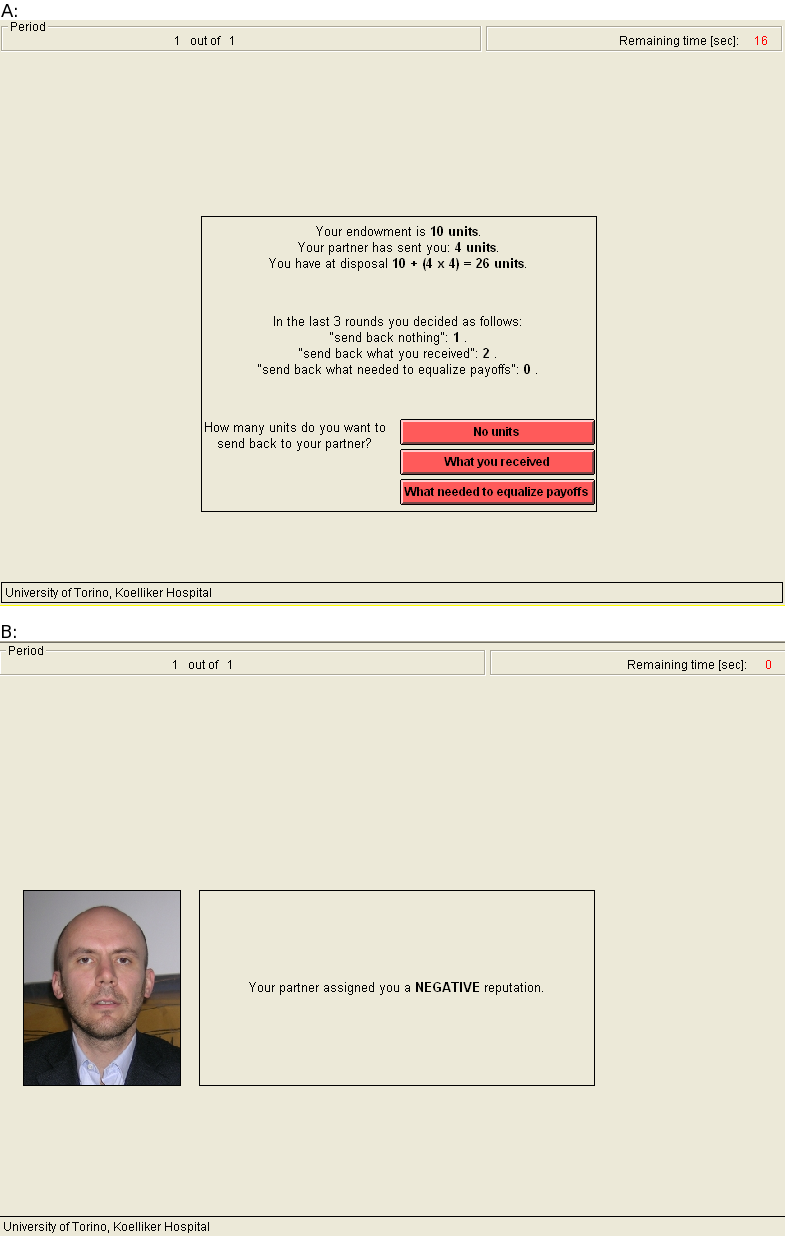

Supplement: Figure S1 — a) Trustee's decision screen (choice phase). b) Trustee's reputation outcome screen (reaction phase). In the reputation treatment, the subject could see along his picture the reputation judgment, either “positive reputation” or “negative reputation”, that he received by the investor. In the no-reputation treatment, under the subject's picture there was the writing: “no reputation has been assigned”. The person in the picture has given written informed consent, as outlined in the PLOS consent form, to publication of his photograph. We also specified that the image used in this figure is not the original image used in the study, but a similar one used for illustrative purposes only. (TIFF) [file pone.0106285.s001.tiff]

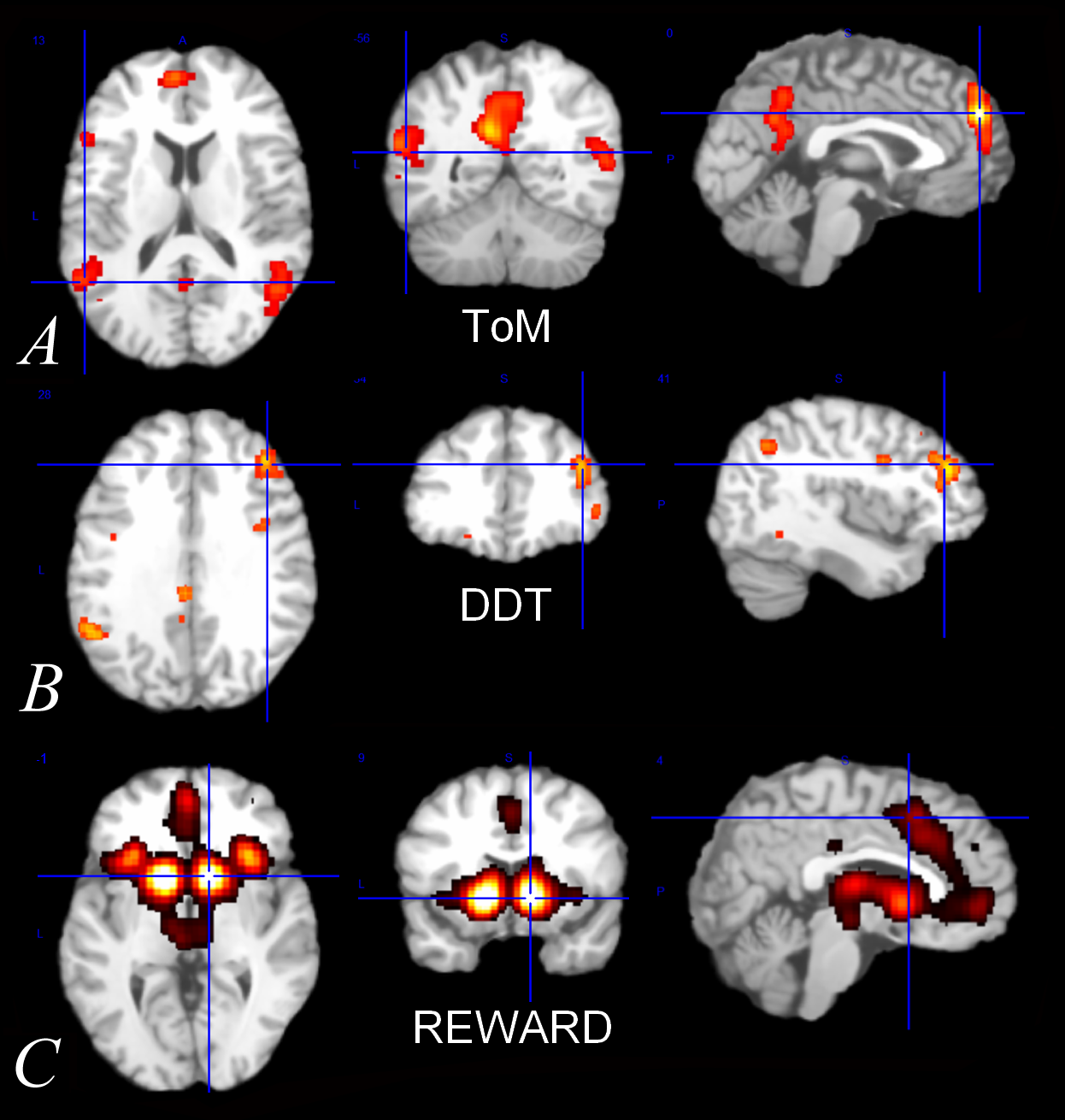

Supplement: Figure S2 — fMRI and PET meta-analysis results. From top to bottom: A) Theory of Mind (TOM) ALE; B) Delay Discounting Task (DDT) ALE; C) Reward ALE. Consistent ALE clusters, using p<0.01 FDR corrected for multiple comparisons, Ke>200 mm3. A = Anterior, P = Posterior, S = Superior, L = Left, R = Right (neurological convention). Statistical maps overlaid onto a Talairach space template. From top to bottom: z = −1 mm, y = 9 mm, x = 4 mm; z = 28 mm, y = 34 mm, x = 41 mm; z = 13 mm, y = −56 mm, x = 0 mm. (TIF) [file pone.0106285.s002.tif]

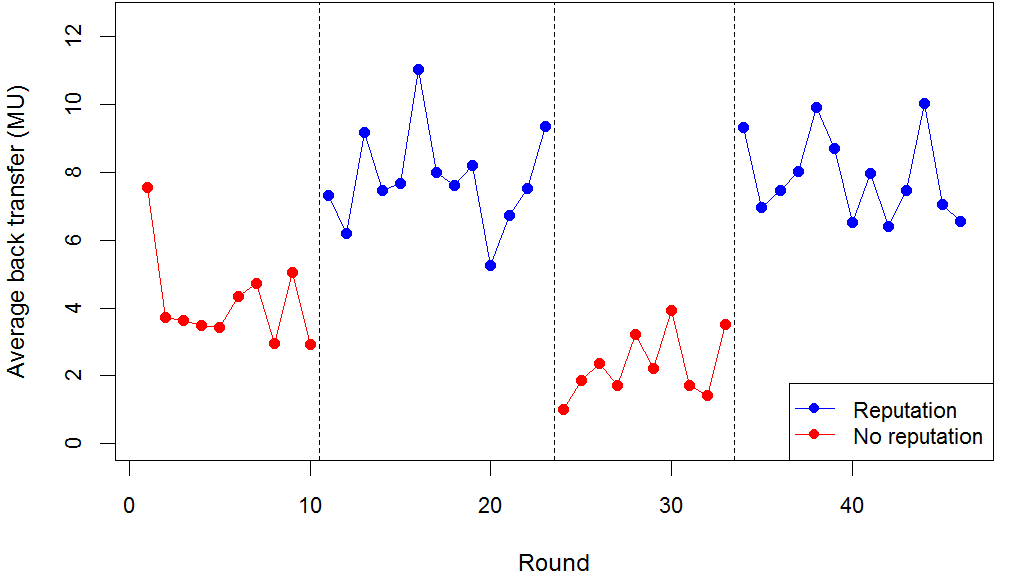

Supplement: Figure S3 — Back transfer dynamics in the game. Vertical dashed lines mark condition changes. (TIFF) [file pone.0106285.s003.tiff]

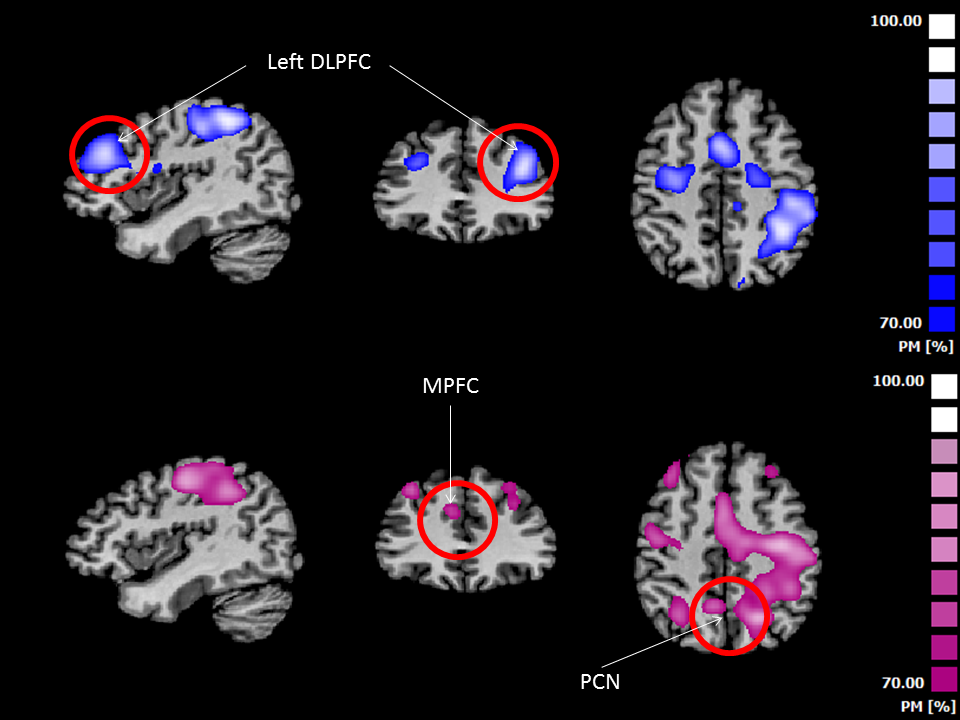

Supplement: Figure S4 — Probability maps of the Males and Females activity in the Reputation > No-Reputation contrast in the Choice phase. From top to bottom: males (in blue) and females (in pink) probability maps of significantly activated clusters, p<0.05 uncorrected. Statistical maps overlaid on a Talairach space template. Radiological convention: right on the left side of the figure. DLPFC = Dorso-Lateral Pre-Frontal Cortex. MPFC = Medial Pre-Frontal Cortex. PCN = Precuneus. (TIF) [file pone.0106285.s004.tif]
