# Supplementary material for: Neural Correlates of Gender Differences in Reputation Building
Source: PLoS One. 2014 Sep 2;9(9):e106285. doi: 10.1371/journal.pone.0106285 (PMC4152267; doi:10.1371/journal.pone.0106285)
Supplement: File S1 — Supplemental Methods and Results. Including: Simulated agents; Matlab script; Preliminary fMRI analysis and results; Path Analysis with Structural Equation Modeling; fMRI and PET Meta-analysis; Supplemental analysis of behavioral data; Supplemental References; List of the references included in the three meta-analyses; Tables S1–S6. (PDF) [file pone.0106285.s005.pdf]

File S1

Supplemental Methods and Results

Simulated agents

Simulated agents behave probabilistically based on the past choices of their partners. Specifically, artificial investors playing the dilemma have to make two decisions: to decide how many units send to the trustee and to evaluate trustee behavior (i.e., they assign reputation to trustees). In the no-reputation treatment, the four artificial investor choices (1, 4, 7, 10 units) are equally likely to occur. In the reputation treatment, artificial investor behavior is based upon probability distributions, trustee reputation and past choices (Tab. S1).

Similarly, when assigning a reputation to a partner, artificial investors look at the three last choices made by the trustee. When choices have been all the same and signaling a specific behavior (i.e., the first two columns of the table, where trustees have repeatedly chosen either to send back nothing or to equalize payoffs), the reputation judgment is deterministic (negative or positive, respectively). When trustee past behavior is less clear, probabilities award cooperative behavior with higher chances of a positive reputation, and vice versa (Tab. S1).

Matlab script

A script (http://precedings.nature.com/documents/6142/version/2) was written in Matlab (The MathWorks, Inc., Natick, MA) to tabulate Talairach coordinates of local maxima of all activation maps and to determine the activated brain regions and Brodmann Areas (BA) according to the Analysis of Functional Neuroimages (AFNI, http://afni.nimh.nih.gov) atlas. The same script was used to compute the lateralization indices L% and R% for each cluster of brain activity, and for anatomical or BA subregions of clusters, according to the following formulae $L = \frac{n_{voxL}}{n_{voxL} + n_{voxR}}$; $R = \frac{n_{voxR}}{n_{voxL} + n_{voxR}}$

, where nvoxL is the number of active voxels in a cluster in the left cerebral hemisphere, and nvoxR is the number of active voxels in the homologous region of the right hemisphere.

Preliminary fMRI analysis and results

In order to investigate the difference between Positive and Negative evaluation in the Reaction phase, we performed a preliminary GLM analysis using a more complex model than that presented in the Main Text (MT), which also included the two levels (Negative or Positive) of the Reaction phase as regressors. However, the contrast between Negative and Positive reputation did not lead to any significant difference in brain activations. This is consistent with Liu et al. (2011) meta-analysis on reward valence, suggesting that some components of the reward network are commonly activated by both positive and negative rewards across various stages of reward processing (e.g., anticipation, outcome and evaluation).

We were unable to perform a GLM including the three behavioral levels of back transfer (nothing; the same amount sent by A; an amount that equalize payoff between A and B) as regressors, because only 7 out of 16 subjects used all the three possible choices during the game. For the other 9 subjects one level, depending on the subject, was missing. Thus, in order to control the behavior effects in our data, we performed the ANCOVA described in the MT.

Path Analysis with Structural Equation Modeling

We investigated whether gender has a significant direct impact on behavior by means of a path analysis conducted with Structural Equation Modeling (SEM).

In particular, we extracted a dataset composed by trial by trial beta values of activation in 8 brain areas. Selected areas are those that, in the ANOVA analysis have been found significantly covariant either with Gender (Caudato and Insuala) or with the interacted variable between Reputation and

Gender (precuneus, fusiform gyrus, DLPFC and DMPFC) in the Choice phase and with Gender (fusiform gyrus, VLPFC) in the Reaction phase. We also included behavioral choices and information about subjects' gender, treatments and runs of the experiment and we obtained an unbalanced dataset with a total of 690 observations for the 16 subjects.

In SEM we consider four exogenous variables: Gender (Male or Female), Reputation (Reputation or No Reputation), the interacted variable between Gender and Reputation and the variable indicating the run of the experiment. Endogenous variables are activation values in the 8 brain areas introduced above and one behavioral variable that represents the choice made by subjects and it is measured as the rate between the units given back to investors and the total endowment available.

We developed a SEM model starting from the following assumptions:

1) all brain areas have a direct effect on behavior, as suggested by the data analysis presented throughout the paper;
2) brain areas can be correlated and this can be modeled only in terms of covariance of error terms (i.e., in the model there cannot be any directed causal relationship between brain areas);
3) any exogenous variable can directly predict any endogenous variable.

Following these hypotheses, a SEM model is developed by means of an iterative procedure lead by a criterion of model fitness. The procedure starts from a random configuration of relationships between variables, then causal links and covariances among error terms of endogenous variables are removed if not significant ($p > 0.05$) and added if their modification index points out a significant improvement ($p < 0.05$) of the model in terms of reduction in its χ^2.

Following the assumptions above, direct effects between brain areas and the behavioral variable are kept constant throughout the procedure. Both covariances between error terms of endogenous variables and causal links directed from exogenous variables to endogenous ones are introduced and kept in the model only if they significantly improve the capability of the model to fit the data. The procedure stops when modifications improving the model further and significantly are not available.

The resulting SEM model does not significantly fail in reproducing the covariance matrix for the 13 variables considered, in fact its χ^2 is small relative to the degrees of freedom and not significant ($p > 0.05$). Moreover, the probability of the model to have a root mean squared error of approximation less than or equal to 0.05 is 0.998 and its Comparative Fit Index is 0.984.

The SEM model showed what follows:

1) exogenous variables (Gender - male or female -, Reputation, - Reputation or No-Reputation , the interacted variable between Gender and Reputation and the variable indicating the run of the experiment) do not have any direct effect on the behavioral variable;
2) there is only one direct effect of the variable indicating the experimental run and it is on the Fusiform gyrus, showing, during the reaction phase, a greater activity in the No-Reputation than in the Reputation treatment;
3) Reputation has a direct effect on PCN, Caudato and VLPFC;
4) Gender has a direct effect on DLPFC, Caudato, Insula, Fusiform (reaction phase) and VLPFC;
5) The interacted variable between Gender and Reputation has a direct effect on Fusiform (choice phase), DLPFC, DMPFC and Caudato;
6) All brain areas have a significant ($p < 0.05$) direct effect on the behavioral variable, except Caudato and Fusiform (reaction phase);
7) The Gender and Reputation variables have a significant indirect effect on the behavioral variable (respectively, $p = 0.005$ and $p = 0.000$).

To sum up, the SEM model showed that gender does not have a significant direct effect on behavior. Nevertheless, gender does have a significant indirect effect on behavior, that is to say that gender predicts behavior only if mediated by brain areas.

Also different models were developed and all of them confirmed the results of the model described above. Results are confirmed when the behavioral variable is measured either as the absolute amount of units given back to investors or as its difference with the average individual amount of restitution

in the baseline treatment. Similarly, again, results are also confirmed when relaxing the first assumption described above. In fact, we developed a further model where direct effects spanning from brain areas to behavior are evolved according to model fitness instead of imposing a constant structure. All these models do not contain a direct effect of gender on behavior.

In all models, estimation of parameters has been conducted with a maximum likelihood algorithm considering missing values (the behavioral variable is missing when subjects did not make a choice in time or pushed the wrong button during the experiment). In all models, the variance-covariance matrix of the estimates has been estimated by means of the generalized Huber-White-Sandwich estimator considering each subject as a single group (i.e., cluster-robust standard errors).

Further details about SEM models are available from authors on request.

fMRI and PET Meta-analysis

We generate three meta-analyses of interest using the BrainMap database, the Sleuth 2.0.3 and GingerAle 2.1 software.

BrainMap (http://www.brainmap.org) is a database of published functional neuroimaging studies (mainly PET and fMRI) that contains both metadata descriptions of experimental design and activation locations in the form of stereotactic coordinates (Fox and Lancaster, 2002). BrainMap contains 2298 neuroimaging papers that analyse 10924 experiments using 100 unique paradigm classes, yielding to 87,683 locations or foci (May 11, 2013).

We extracted from the BrainMap database all the studies involving only normal subjects that reported an activation in three Experimental Paradigm Classes of interest described below:

Reward Task	a behavioral experimental paradigm in which, in at least one of the conditions, subjects perform a task in which correct performance is associated with reward, often monetary reward;

Delay Discounting Task	a behavioral experimental paradigm that measures subject self-control, i.e. the capacity for resist the temptation of an immediately delivered small reward, in order to obtain a large reward delivered at variable delays;
Theory of Mind Task	a behavioral experimental paradigm in which the subjects should attribute mental states - beliefs, intents, desires, pretending, knowledge, etc. - to himself and others and to understand others beliefs, desires and intentions as different from their own.

We sent queries to the database with the Sleuth 2.0.3 software (Fox et al., 2005). The specific queries were:

[Diagnosis = Normals] AND [Experiment Paradigm Class = Reward Task];

[Diagnosis = Normals] AND [Experiment Paradigm Class = Delayed Discount Task];

[Diagnosis = Normals] AND [Experiment Paradigm Class = Theory of Mind Task].

The results were:

Reward Task	169 papers (159 fMRI, 10 PET), 3378 subjects, 838 experiments, 6274 foci;
Delay Discounting Task	11 papers (11 fMRI), 352 subjects, 50 experiments, 651 foci;
Theory of Mind Task	26 papers (22 fMRI, 4 PET), 530 subjects, 121 experiments, 828 foci.

The Activation Likelihood Estimation (ALE) analysis is a quantitative method that can be used to estimate consistent activation across different imaging studies (Eickhoff *et al*., 2009). ALE maps of coactivations are derived based on patterns of foci of interest, where multiple studies have reported statistically significant peak activation. To limit intersubject and interlaboratory variability, we used an algorithm that estimates the spatial uncertainty of each focus, taking into account the possible

differences among the neuroimaging studies (Eickhoff *et al*., 2009). This algorithm was preferred to a specified FWHM as in the original ALE approach. The advantage of such an algorithm is that it limits the meta-analysis to an anatomically constrained space specified by a gray matter task. Furthermore, it comprises a method to calculate the above-chance clustering between experiments (i.e., random effects analysis) rather than between foci (fixed effects analysis). Regions of convergence were calculated using GingerAle 2.1 software (Eickhoff *et al*., 2009; Eickhoff *et al*., 2012) in the Talairach space (Talairach and Tournoux, 1988) with the more conservative mask size and FWHM values subject-based. The p threshold was False Discovery Rate with positive dependence assumption, FDR pN < 0.01 and minimum clusters extent Ke > 200 mm^3. We used Chris Rorden's MRIcron software (http://www.nitrc.org/projects/mricron) for visualize and save images, overlaying the GingerAle maps onto a Talairach brain template (created by Kochunov *et al*., 2002). See below the list of references included in the three meta-analyses and see results in Tab. S2 and Fig. S2.

Supplemental analysis of behavioral data

Game dynamics

The game dynamic showed a systematic increase of back transfers in the Reputation treatment rounds (Fig. S3). In both runs, the reputation conditions led to similar averages (7.86 ± 0.54 MU vs. 7.81 ± 0.52 MU, V= 44, p = 0.112 one tailed) while the no reputation condition of the second run led to significantly lower back transfers than the no reputation condition of the first run (4.10 ± 0.53 MU vs. 2.29 ± 0.38 MU, V= 19, p = 0.011 one tailed). It is also worth noting that, immediately after the change from the no reputation to the reputation condition, subjects increased their back transfer. On average back transfer in the last no reputation round were 3.23 ± 0.77 MU and became 8.29 ± 1.24 MU in the first reputation round. This difference is highly significant (V= 114.5, p = 0.009). Back transfers further increased when subjects started to receive evaluations for their

choices, rising from 7.77 ± 0.80 MU in the first three rounds of both reputation conditions to 8.78 ± 0.81 MU in the subsequent three rounds. However, this difference is not significant (V= 83, p = 0.101).

Behavioral effects of the received evaluations

Overall subjects received a total of 288 evaluations, with a majority of positive ones (163 positive vs. 126 negative). During the second reputation condition, the proportion of positive evaluations increased from 53.5% to 59.7%. Subjects strongly reacted to the evaluations received, showing a clearly increasing trend of back transfers following a negative evaluation, while decreasing them after a positive one (see MT, Fig. 2B).

While in the no reputation condition the trend was to decrease back transfers over time (on average, by 0.52 ± 0.46 MU per round), in the reputation condition the increase or decrease of this amount depended on the evaluation received in the previous round. More precisely, in the first three rounds of the reputation condition, when no evaluation was yet communicated, participants on average increased their back transfer by 1.19 ± 0.97 MU per round. Subsequently, they increased average back transfers after a negative evaluation (2.68 ± 0.76 MU) and decreased them after a positive evaluation (−1.98 ± 0.79 MU). The differences with the first three rounds of the reputation condition, where no evaluation took place due to insufficient past data, is significant for both negative and positive evaluations (V = 30, p = 0.025 one tailed, and V = 111, p = 0.002 one tailed, respectively). Differences with the baseline are highly significant for negative evaluations (V = 13, p = 0.001 one tailed) but only significant at the 10% level for positive evaluations (V = 98, p = 0.065 one tailed).

These results are confirmed by a fixed effects model using the first order differences in back transfer as dependent and the evaluations received (in comparison with the no reputation treatment) as independents, controlled by the amount sent by A players. All coefficients are highly significant except the one related to the condition where subjects did not yet receive an evaluation, which is

significant only at the 10% level. Consistent with the analysis above, subjects increased their back transfer following a negative evaluation, but decreased them following a positive one (Tab. S3).

Payoffs

Average payoffs were slightly lower in the reputation rounds (25.4 ± 0.6 MU/round) than in the baseline ones (29.9 ± 0.7 MU/round) due to the fact that B players who transferred back a higher share of their endowment and this was not compensated by the higher amounts sent by the artificial agents playing in the A position. The difference is significant at the 1% level (V = 18, p = 0.004 one tailed). Gender differences in payoffs are not significant neither in the baseline nor in the reputation rounds (Baseline: females 28.2 ± 1.0 MU/round, males 29.5 ± 1.1 MU/round, W = 24, p = 0.221 one tailed; Reputation treatment: females 25.2 ± 0.7 MU/round, males 25.5 ± 0.9 MU/round, W = 33, p = 0.489 one tailed).

References

Eickhoff SB, Bzdok D, Laird AR, Kurth F, Fox PT (2012) Activation likelihood estimation revisited. Neuroimage, 59, 2349-2361.

Eickhoff SB, Laird AR, Grefkes C, Wang LE, Zilles K, Fox PT (2009) Coordinate-based activation likelihood estimation meta-analysis of neuroimaging data: A random-effects approach based on empirical estimates of spatial uncertainty. Hum Brain Mapp, 30, 2907-2926.

Fox PT, Lancaster JL (2002) Mapping context and content: The BrainMap model. Nature Rev Neurosci, 3, 319-321.

Fox PT, Laird AR, Fox SP, Fox PM, Uecker AM, Crank M, Koenig SF, Lancaster JL (2005) BrainMap taxonomy of experimental design: Description and evaluation. Hum Brain Mapp, 25, 185-198.

Kochunov P, Lancaster J, Thompson P, Toga AW, Brewer P, Hardies J, Fox P (2002) An optimized individual target brain in the Talairach coordinate system. Neuroimage, 17, 922-7.

Liu X, Hairston J, Schrier M, Fan J (2011) Common and distinct networks underlying reward valence and processing stages: a meta-analysis of functional neuroimaging studies. Neurosci Biobehav Rev. 35(5):1219-36

Talairach J & Tournoux L (1988) Co-planar stereotaxic atlas of the human brain: 3-dimensional proportional system — an approach to cerebral imaging. New York: Thieme.

Table S1: Simulated agents' behavior. From top to bottom: **A**) The Investor's choice probabilities in the reputation treatment; **B**) The distribution of the past three actions of the trustee; **C**) The probability of receiving a positive or negative evaluation (depending on B); **D**) The combination of the Investor's and the Trustee's choices.

**A**	**Trustee's reputation**		
	**Units**	**Positive**	**Negative**
**Investor's choice**	**1**	0.05	0.45
	**4**	0.15	0.35
	**7**	0.35	0.15
	**10**	0.45	0.05

**B**	**Trustee's three past choices**									
**Send back nothing**	0	3	1	0	0	0	1	1	2	2
**Send back as received**	0	0	1	3	1	2	0	2	0	1
**Equalize payoffs**	3	0	1	0	2	1	2	0	1	0

**C**	**Probability of receiving an evaluation**									
**Positive**	1	0	0.5	0.5	0.8	0.6	0.7	0.4	0.3	0.2
**Negative**	0	1	0.5	0.5	0.2	0.4	0.3	0.6	0.7	0.8

**D**		**Trustee's choice**					
		**Send back nothing**		**Send back as received**		**Equalize payoffs**	
**Investor's choice**		Investor	Trustee	Investor	Trustee	Investor	Trustee
**Units**	1	9	14	10	13	11.5	11.5
	4	6	26	10	22	16	16
	7	3	38	10	31	20.5	20.5
	10	0	50	10	40	25	25

Table S2: fMRI and PET meta-analysis results.

From top to bottom: Theory of Mind ALE; Delay Discounting Task ALE; Reward ALE.

Cluster	Brain Region	Ke	X	y	Z	Max ALE
**THEORY OF MIND ALE**						
1	L Superior Frontal Gyrus (BA9)	8968	-2	48	30	0.065
	L Medial Frontal Gyrus (BA9)		-4	50	18	0.039
	L Superior Frontal Gyrus (BA9)		-24	46	36	0.036
2	L Posterior Cingulate (BA31)	6272	-8	-54	24	0.050
	R Cingulate Gyrus (BA31)		2	-60	28	0.034
	R Precuneus (BA7)		4	-54	38	0.033
3	R Middle Occipital Gyrus (BA37)	6024	48	-68	6	0.041
	R Middle Temporal Gyrus (BA19)		48	-60	16	0.034
	R Superior Temporal Gyrus (BA22)		52	-50	12	0.029
	R Superior Temporal Gyrus (BA22)		48	-36	2	0.026
4	L Superior Temporal Gyrus (BA22)	3808	-54	-56	18	0.035
	L Middle Temporal Gyrus (BA37)		-42	-64	8	0.027
5	L Middle Temporal Gyrus (BA 22)	2464	-56	-34	-2	0.033
	L Superior Temporal Gyrus (BA22)		-48	-36	2	0.030
6	R Middle Temporal Gyrus (BA21)	1704	56	-10	-12	0.037
**DELAY DISCOUNTING TASK ALE**						
1	L Inferior Parietal Lobule (BA 40)	4664	-34	-54	42	0.035
	L Supramarginal Gyrus (BA 40)		-54	-52	30	0.033
2	R Middle Frontal Gyrus (BA 10)	3208	40	36	24	0.035
3	R Fusiform Gyrus (BA 37)	2824	48	-54	-10	0.037
	R Inferior Temporal Gyrus (BA 20)		52	-28	-12	0.022
	R Middle Temporal Gyrus (BA 21)		58	-20	-16	0.020
4	R Angular Gyrus (BA 39)	2592	42	-58	36	0.027

	R Superior Parietal Lobule (BA 7)		30	-60	46	0.027
	R Inferior Parietal Lobule (BA 40)		38	-42	42	0.021
5	L Limbic Lobe.Cingulate Gyrus (BA 31)	1808	-2	-34	30	0.031
6	L Caudate Head	1448	-8	4	-2	0.038
	L Medial Globus Pallidus		-14	-6	-4	0.020
7	L Claustrum	1384	-24	16	6	0.024
	L Caudate Body		-14	-2	18	0.023
	L Caudate Body		-18	12	14	0.022
8	L Middle Frontal Gyrus (BA 10)	1304	-38	44	-2	0.023
	L Inferior Frontal Gyrus (BA 45)		-46	36	2	0.018
9	R Middle Frontal Gyrus (BA 8)	1280	38	22	44	0.023
	R Superior Frontal Gyrus (BA 6)		26	12	48	0.023
10	L Middle Temporal Gyrus (BA 21)	1152	-60	-28	-4	0.022
11	L Inferior Occipital Gyrus (BA 17)	1088	-14	-88	-6	0.037
12	R Putamen	1024	24	16	-8	0.031
**REWARD ALE**						
1	L Lateral Globus Pallidus	117416	-12	8	0	0.450
	R Caudate Head		12	8	0	0.429
	R Insula		32	18	2	0.270
	L Claustrum		-30	18	2	0.247
	R Thalamus		2	-16	10	0.194
	L Anterior Cingulate Cortex (BA32)		0	48	-2	0.181
	R Amygdala		22	-2	-10	0.162
	R Medial Frontal Gyrur (BA6)		2	14	44	0.150
	L Cingulate Gyrus (BA32)		0	24	34	0.149
	L Cingulate Gyrus (BA24)		-2	2	46	0.126

2	L Superior Parietal Lobule (BA7)	2432	-28	-62	42	0.107
	L Superior Parietal Lobule (BA7)		-30	-58	42	0.107
3	L Cingulate Gyrus (BA23)	2384	0	-28	32	0.129
4	R Middle Frontal Gyrus (BA10)	1520	34	48	2	0.082
5	L Middle Frontal Gyrus (BA10)	1096	-32	44	18	0.088
6	R Inferior Parietal Lobule (BA40)	1392	40	-54	48	0.080
	R Inferior Parietal Lobule (BA40)		42	-46	44	0.075
	R Superior Parietal Lobule (BA7)		24	-64	40	0.092

Consistent ALE clusters, $p<0.01$, FDR corrected for multiple comparisons. Ke = cluster extension > 1000 mm^3. (BA) = Brodmann Area. L = Left. R = Right. x, y, z expressed in mm. Coordinates were reported in Talairach space. Brain regions are classified using Talairach Daemon Tool (http://www.talairach.org/daemon.html).

Table S3: Fixed effect model on back transfer differences.

	Estimate	S.E.	t	p
Previous evaluation: Negative	3.124	0.920	3.394	0.001
Previous evaluation: None	1.623	0.924	1.756	0.080
Previous evaluation: Positive	–2.070	0.847	–2.443	0.015
Amount sent	0.911	0.096	9.509	0.000
R^2	0.150			
F(4,660)	29.093			<0.001

Table S4: Single subjects analysis. Percentage of males and females with significantly activation in peak areas of the Interaction Gender x Reputation during Choice Phase (see Table 1, in MT).

**Peak Area (Brodmann Area)**	**x**	**y**	**Z**	**Males %**	**Females %**
R Medial Frontal Gyrus (BA 8)	11	37	42	0%	54%
L Precuneus (BA 7)	-13	-53	45	13%	88%
L Fusiform Gyrus (BA 37)	-31	-32	-21	13%	63%
L Middle Frontal Gyrus (BA 9, 46)	-37	37	24	100%	30%

Significantly activated clusters, $p<0.05$, uncorrected. L = Left. R = Right. x, y, z expressed in mm. Coordinates were reported in Talairach space. Brain regions are classified using AFNI (http://afni.nimh.nih.gov) atlas.

Table S5: ANCOVA results for the Choice phase. The results of the ANCOVA, with a "Behavior" covariate, is shown in the upper part of the Table for the areas showing a Gender effect and in the lower part for the areas showing an Interaction Gender*Reputation effect (see Table 1 in the MT).

GENDER (MALES > FEMALES)							
	Degr. of freedom	F	p	Partial eta-squared	Power (alpha=0.05)	Clust.	Brain region
Intercept	1	17.55582	0.001059	0.574549	0.971567	1	**R Crb [Crus 1]**
BEHAVIOR	1	3.26304	0.094051	0.200641	0.387307		
GENDER	1	31.28832	0.000087	0.706469	0.999279		
Error	13						
TREATMENT	1	7.96185	0.014419	0.379826	0.741797		
TREATMENT*BEHAVIOR	1	1.90013	0.191324	0.127524	0.248181		
TREATMENT*GENDER	1	1.19116	0.294910	0.083937	0.173112		
Error	13						
Intercept	1	4.31029	0.058280	0.249002	0.484975	**2**	**R Insula**
BEHAVIOR	1	9.87340	0.007788	0.431654	0.827295		
GENDER	1	31.22576	0.000088	0.706054	0.999266		
Error	13						
TREATMENT	1	9.14638	0.009771	0.412997	0.798196		
TREATMENT*BEHAVIOR	1	0.00026	0.987382	0.000020	0.050026		
TREATMENT*GENDER	1	0.36856	0.554247	0.027569	0.087090		
Error	13						
Intercept	1	12.68022	0.003483	0.493774	0.907901	3	**R Midd. Frontal Gyrus**
BEHAVIOR	1	2.23751	0.158575	0.146842	0.283528		
GENDER	1	17.05328	0.001186	0.567435	0.967774		
Error	13						
TREATMENT	1	12.96453	0.003228	0.499317	0.913772		
TREATMENT*BEHAVIOR	1	1.20763	0.291737	0.084999	0.174857		
TREATMENT*GENDER	1	2.80299	0.117964	0.177371	0.341576		
Error	13						
Intercept	1	31.04183	0.000090	0.704826	0.999227	4	**R Cingulate Gyrus**
BEHAVIOR	1	0.01376	0.908410	0.001057	0.051360		
GENDER	1	20.55707	0.000561	0.612600	0.986758		
Error	13						
TREATMENT	1	1.78039	0.205004	0.120456	0.235549		
TREATMENT*BEHAVIOR	1	1.91272	0.189955	0.128261	0.249508		
TREATMENT*GENDER	1	0.00623	0.938299	0.000479	0.050615		
Error	13						
Intercept	1	31.98857	0.000079	0.711038	0.999408	5	**L Caudate**
BEHAVIOR	1	14.90421	0.001968	0.534121	0.945498		
GENDER	1	69.64669	0.000001	0.842704	1.000000		
Error	13						
TREATMENT	1	1.44878	0.250184	0.100270	0.200430		
TREATMENT*BEHAVIOR	1	0.10119	0.755456	0.007724	0.060050		

TREATMENT*GENDER	1	0.33128	0.574733	0.024850	0.083282		
Error	13						
Intercept	1	25.33032	0.000229	0.660843	0.996261	6	**L Midd. Frontal Gyrus**
BEHAVIOR	1	0.07135	0.793570	0.005459	0.057074		
GENDER	1	14.30674	0.002282	0.523927	0.937121		
Error	13						
TREATMENT	1	2.52491	0.136076	0.162636	0.313251		
TREATMENT*BEHAVIOR	1	0.33976	0.569939	0.025470	0.084148		
TREATMENT*GENDER	1	4.61455	0.051129	0.261974	0.511456		
Error	13						
**INTERACTION GENDER X REPUTATION**							
	Degr. of	F	p	Partial eta-squared	Observed power (alpha=0.05)	Clust.	Brain region
Intercept	1	0.02105	0.886869	0.001617	0.052081	1	**L Medial Frontal Gyr.**
BEHAVIOR	1	2.03393	0.177394	0.135289	0.262247		
GENDER	1	0.56703	0.464860	0.041795	0.107538		
Error	13						
TREATMENT	1	0.60281	0.451405	0.044315	0.111251		
TREATMENT*BEHAVIOR	1	0.90754	0.358146	0.065255	0.143143		
TREATMENT*GENDER	1	28.13809	0.000143	0.683991	0.998264		
Error	13						
Intercept	1	2.40268	0.145122	0.155991	0.300660	**2**	**L Precuneus**
BEHAVIOR	1	0.54207	0.474654	0.040028	0.104951		
GENDER	1	0.00179	0.966880	0.000138	0.050177		
Error	13						
TREATMENT	1	7.22662	0.018607	0.357283	0.700603		
TREATMENT*BEHAVIOR	1	0.95715	0.345761	0.068578	0.148371		
TREATMENT*GENDER	1	20.84371	0.000530	0.615881	0.987706		
Error	13						
Intercept	1	3.43361	0.086713	0.208938	0.403858	3	**L Fusiform Gyrus**
BEHAVIOR	1	5.19855	0.040116	0.285658	0.559719		
GENDER	1	10.27461	0.006895	0.441451	0.841730		
Error	13						
TREATMENT	1	0.35733	0.560260	0.026752	0.085943		
TREATMENT*BEHAVIOR	1	0.00076	0.978453	0.000058	0.050075		
TREATMENT*GENDER	1	5.06290	0.042400	0.280293	0.548814		
Error	13						
Intercept	1	34.45283	0.000055	0.726044	0.999706	4	**L Midd. Frontal Gyrus**
BEHAVIOR	1	1.25284	0.283266	0.087901	0.179649		
GENDER	1	4.46508	0.054503	0.255658	0.498559		
Error	13						
TREATMENT	1	11.34862	0.005036	0.466089	0.875258		
TREATMENT*BEHAVIOR	1	1.66467	0.219452	0.113516	0.223312		
TREATMENT*GENDER	1	18.27873	0.000904	0.584382	0.976287		
Error	13						

Table S6: ANCOVA results for the Reaction phase. The results of the ANCOVA, with a "Behavior" covariate, is shown for the areas showing a Gender effect, in the upper part Males>Females; in the lower part Females>Males (see Table 2 in the MT).

GENDER FACTOR – MALES > FEMALES							
	Degr. of freedom	F	p	Partial eta-squared	Power (alpha=0.05)	Clust.	Brain region
Intercept	1	21.69164	0.000448	0.625270	0.990146	1	**L Inf. Front. Gyr.**
BEHAVIOR	1	0.57494	0.461830	0.042353	0.108357		
GENDER	1	16.34822	0.001394	0.557043	0.961640		
Error	13						
TREATMENT	1	0.19371	0.667072	0.014682	0.069332		
TREATMENT*BEHAVIOR	1	0.07655	0.786384	0.005854	0.057592		
TREATMENT*GENDER	1	0.81696	0.382513	0.059128	0.133621		
Error	13						
**GENDER FACTOR - FEMALES > MALES**							
	Degr. of freedom	F	p	Partial eta-squared	Power (alpha=0.05)	Cluster	Brain region
Intercept	1	73.74539	0.000001	0.850136	1.000000	1	**R Fusiform Gyrus**
BEHAVIOR	1	0.36636	0.555415	0.027409	0.086865		
GENDER	1	20.53487	0.000564	0.612344	0.986681		
Error	13						
TREATMENT	1	3.62227	0.079388	0.217917	0.421887		
TREATMENT*BEHAVIOR	1	0.14196	0.712419	0.010802	0.064129		
TREATMENT*GENDER	1	0.81804	0.382209	0.059201	0.133734		
Error	13						
Intercept	1	64.14865	0.000002	0.831494	1.000000	2	**L Sup. Par. Gyr.**
BEHAVIOR	1	13.22499	0.003013	0.504290	0.918847		
GENDER	1	45.18248	0.000014	0.776565	0.999987		
Error	13						
TREATMENT	1	4.65937	0.050166	0.263847	0.515281		
TREATMENT*BEHAVIOR	1	0.00023	0.988174	0.000018	0.050023		
TREATMENT*GENDER	1	0.01522	0.903716	0.001169	0.051504		
Error	13						

List of the references included in the three meta-analyses

REWARD TASK META-ANALYSIS

1. Peters J, Büchel C (2010) Episodic future thinking reduces reward delay discounting through an enhancement of prefrontal-mediotemporal interactions. Neuron 66: 138–148. Available: http://www.ncbi.nlm.nih.gov/entrez/query.fcgi?cmd=Retrieve&db=PubMed&list_uids=20399735&dopt=Abstract. Accessed 10 March 2014.

2. Abler B, Erk S, Walter H (2007) Human reward system activation is modulated by a single dose of olanzapine in healthy subjects in an event-related, double-blind, placebo-controlled fMRI study. Psychopharmacology (Berl) 191: 823–833. Available: http://www.ncbi.nlm.nih.gov/entrez/query.fcgi?cmd=Retrieve&db=PubMed&list_uids=17265148&dopt=Abstract.

3. Abler B, Walter H, Erk S, Kammerer H, Spitzer M (2006) Prediction error as a linear function of reward probability is coded in human nucleus accumbens. Neuroimage 31: 790–795. Available: http://www.ncbi.nlm.nih.gov/entrez/query.fcgi?cmd=Retrieve&db=PubMed&list_uids=16487726&dopt=Abstract.

4. Adcock RA, Thangavel A, Whitfield-Gabrieli S, Knutson B, Gabrieli JDE (2006) Reward-motivated learning: Mesolimbic activation precedes memory formation. Neuron 50: 507–517. Available: http://www.ncbi.nlm.nih.gov/entrez/query.fcgi?cmd=Retrieve&db=PubMed&list_uids=16675403&dopt=Abstract.

5. Akitsuki Y, Sugiura M, Watanabe J, Yamashita K, Sassa Y, et al. (2003) Context-dependent cortical activation in response to financial reward and penalty: An event-related fMRI study. Neuroimage 19: 1674–1685. Available: http://www.ncbi.nlm.nih.gov/entrez/query.fcgi?cmd=Retrieve&db=PubMed&list_uids=12948722&dopt=Abstract.

6. Alexander WH, Brown JW (2010) Competition between learned reward and error outcome predictions in anterior cingulate cortex. Neuroimage 49: 3210–3218. Available:

http://www.ncbi.nlm.nih.gov/entrez/query.fcgi?cmd=Retrieve&db=PubMed&list_uids=19961940&dopt=Abstract. Accessed 17 March 2014.

7. Beck A, Schlagenhauf F, Wustenberg T, Hein J, Kienast T, et al. (2009) Ventral striatal activation during reward anticipation correlates with impulsivity in alcoholics. Biol Psychiatry 66: 734–742. Available: http://www.ncbi.nlm.nih.gov/entrez/query.fcgi?cmd=Retrieve&db=PubMed&list_uids=19560123&dopt=Abstract.

8. Behrens TEJ, Woolrich M, Walton ME, Rushworth MF (2007) Learning the value of information in an uncertain world. Nat Neurosci 10: 1214–1221. Available: http://www.ncbi.nlm.nih.gov/entrez/query.fcgi?cmd=Retrieve&db=PubMed&list_uids=17676057&dopt=Abstract.

9. Bjork JM, Hommer DW (2007) Anticipating instrumentally obtained and passively-received rewards: A factorial fMRI investigation. Behav Brain Res 177: 165–170. Available: http://www.ncbi.nlm.nih.gov/entrez/query.fcgi?cmd=Retrieve&db=PubMed&list_uids=17140674 &dopt=Abstract.

10. Bjork JM, Knutson B, Fong GW, Caggiano DM, Bennett SM, et al. (2004) Incentive-elicited brain activation in adolescents: Similarities and differences from young adults. J Neurosci 24: 1793–1802. Available: http://www.ncbi.nlm.nih.gov/entrez/query.fcgi?cmd=Retrieve&db=PubMed&list_uids=14985419 &dopt=Abstract.

11. Bjork JM, Knutson B, Hommer DW (2008) Incentive-elicited striatal activation in adolescent children of alcoholics. Addiction 103: 1308–1319. Available: http://www.ncbi.nlm.nih.gov/entrez/query.fcgi?cmd=Retrieve&db=PubMed&list_uids=18851716 &dopt=Abstract.

12. Blair K, Marsh AA, Morton J, Vythilingam M, Jones M, et al. (2006) Choosing the lesser of two evils, the better of two goods: Specifying the roles of ventromedial prefrontal cortex and dorsal anterior cingulate in object choice. J Neurosci 26: 11379–11386. Available: http://www.ncbi.nlm.nih.gov/entrez/query.fcgi?cmd=Retrieve&db=PubMed&list_uids=17079666&dopt=Abstract.

13. Bolla KI, Eldreth DA, Matochik JA, Cadet JL (2005) Neural substrates of faulty decision-making in abstinent marijuana users. Neuroimage 26: 480–492. Available: http://www.ncbi.nlm.nih.gov/entrez/query.fcgi?cmd=Retrieve&db=PubMed&list_uids=15907305&dopt=Abstract.

14. Bolla KI, Eldreth DA, Matochik JA, Cadet JL (2004) Sex-related differences in a gambling task and its neurological correlates. Cereb Cortex 14: 1226–1232. Available: http://www.ncbi.nlm.nih.gov/entrez/query.fcgi?cmd=Retrieve&db=PubMed&list_uids=15142963&dopt=Abstract.

15. Boorman ED, Behrens TEJ, Woolrich MW, Rushworth MF (2009) How green is the grass on the other side? Frontopolar cortex and the evidence in favor of alternative courses of action. Neuron 62: 733–743. Available: http://www.ncbi.nlm.nih.gov/entrez/query.fcgi?cmd=Retrieve&db=PubMed&list_uids=19524531&dopt=Abstract.

16. Bray S, Rangel A, Shimojo S, Balleine B, O'Doherty JP (2008) The neural mechanisms underlying the influence of Pavlovian cues on human decision making. J Neurosci 28: 5861–5866. Available: http://www.ncbi.nlm.nih.gov/entrez/query.fcgi?cmd=Retrieve&db=PubMed&list_uids=18509047&dopt=Abstract.

17. Breiter HC, Aharon I, Kahneman D, Dale AM, Shizgal P (2001) Functional imaging of neural responses to expectancy and experience of monetary gains and losses. Neuron 30: 619–639. Available: http://www.ncbi.nlm.nih.gov/entrez/query.fcgi?cmd=Retrieve&db=PubMed&list_uids=11395019&dopt=Abstract.

18. Brunia CHM, de Jong BM, Van den Berg-Lenssen MMC, Paans AMJ (2000) Visual feedback about time estimation is related to a right hemisphere activation measured by PET. Exp Brain Res 130: 328–337. Available: http://www.ncbi.nlm.nih.gov/entrez/query.fcgi?cmd=Retrieve&db=PubMed&list_uids=10706432 &dopt=Abstract.

19. Budhani S, Marsh AA, Pine DS, Blair RJR (2007) Neural correlates of response reversal: Considering acquisition. Neuroimage 34: 1754–1765. Available:

http://www.ncbi.nlm.nih.gov/entrez/query.fcgi?cmd=Retrieve&db=PubMed&list_uids=17188518&dopt=Abstract.

20. Burger KS, Stice E (2012) Frequent ice cream consumption is associated with reduced striatal response to receipt of an ice cream-based milkshake. Am J Clin Nutr 95: 810–817. Available: http://www.ncbi.nlm.nih.gov/entrez/query.fcgi?cmd=Retrieve&db=PubMed&list_uids=22338036&dopt=Abstract. Accessed 17 March 2014.

21. Burke CJ, Tobler PN, Schultz W, Baddeley M (2010) Striatal BOLD Response Reflects the Impact of Herd Information on Financial Decisions. Front Hum Neurosci 4: 48. Available: http://www.ncbi.nlm.nih.gov/entrez/query.fcgi?cmd=Retrieve&db=PubMed&list_uids=20589242&dopt=Abstract. Accessed 10 March 2014.

22. Camara E, Rodriguez-Fornells A, Munte TF (2009) Functional connectivity of reward processing in the brain. Front Hum Neurosci 2: 1–14. Available: http://www.ncbi.nlm.nih.gov/entrez/query.fcgi?cmd=Retrieve&db=PubMed&list_uids=19242558&dopt=Abstract.

23. Chandrasekhar P V, Capra CM, Moore S, Noussair C, Berns GS (2008) Neurobiological regret and rejoice functions for aversive outcomes. Neuroimage 39: 1472–1484. Available: http://www.ncbi.nlm.nih.gov/entrez/query.fcgi?cmd=Retrieve&db=PubMed&list_uids=18042401&dopt=Abstract.

24. Chib VS, Rangel A, Shimojo S, O'Doherty JP (2009) Evidence for a common representation of decision values for dissimilar goods in human ventromedial prefrontal cortex. J Neurosci 29: 12315–12320. Available: http://www.ncbi.nlm.nih.gov/entrez/query.fcgi?cmd=Retrieve&db=PubMed&list_uids=19793990&dopt=Abstract.

25. Christopoulos GI, Tobler PN, Bossaerts P, Dolan RJ, Schultz W (2009) Neural correlates of value, risk, and risk aversion contributing to decision making under risk. J Neurosci 29: 12574–12583. Available: http://www.ncbi.nlm.nih.gov/entrez/query.fcgi?cmd=Retrieve&db=PubMed&list_uids=19812332&dopt=Abstract.

26. Clark L, Lawrence AJ, Astley-Jones F, Gray N (2009) Gambling near-misses enhance motivation to gamble and recruit win-related brain circuitry. Neuron 61: 481–490. Available: http://www.ncbi.nlm.nih.gov/entrez/query.fcgi?cmd=Retrieve&db=PubMed&list_uids=19217383&dopt=Abstract.

27. Cohen MX, Elger CE, Weber B (2008) Amygdala tractography predicts functional connectivity and learning during feedback-guided decision-making. Neuroimage 39: 1396–1407. Available: http://www.ncbi.nlm.nih.gov/entrez/query.fcgi?cmd=Retrieve&db=PubMed&list_uids=17997112&dopt=Abstract.

28. Cooper JC, Hollon NG, Wimmer GE, Knutson B (2009) Available alternative incentives modulate anticipatory nucleus accumbens activation. Soc Cogn Affect Neurosci 4: 409–416. Available: http://www.ncbi.nlm.nih.gov/entrez/query.fcgi?cmd=Retrieve&db=PubMed&list_uids=19843618&dopt=Abstract.

29. Cooper JC, Knutson B (2008) Valence and salience contribute to nucleus accumbens activation. Neuroimage 39: 538–547. Available: http://www.ncbi.nlm.nih.gov/entrez/query.fcgi?cmd=Retrieve&db=PubMed&list_uids=17904386&dopt=Abstract.

30. Coricelli G, Critchley HD, Joffily M, O’Doherty JP, Sirigu A, et al. (2005) Regret and its avoidance: A neuroimaging study of choice behavior. Nat Neurosci 8: 1255–1262. Available: http://www.ncbi.nlm.nih.gov/entrez/query.fcgi?cmd=Retrieve&db=PubMed&list_uids=16116457&dopt=Abstract.

31. Cox SML, Andrade A, Johnsrude IS (2005) Learning to like: A role for human orbitofrontal cortex in conditioned reward. J Neurosci 25: 2733–2740. Available: http://www.ncbi.nlm.nih.gov/entrez/query.fcgi?cmd=Retrieve&db=PubMed&list_uids=15758183&dopt=Abstract.

32. Critchley HD, Mathias CJ, Dolan RJ (2001) Neural activity in the human brain relating to uncertainty and arousal during anticipation. Neuron 29: 537–545. Available: http://www.ncbi.nlm.nih.gov/entrez/query.fcgi?cmd=Retrieve&db=PubMed&list_uids=11239442&dopt=Abstract.

33. D'Ardenne K, McClure SM, Nystrom LE, Cohen JD (2008) BOLD responses reflecting dopaminergic signals in the human ventral tegmental area. Science (80-) 319: 1264–1267. Available: http://www.ncbi.nlm.nih.gov/entrez/query.fcgi?cmd=Retrieve&db=PubMed&list_uids=18309087&dopt=Abstract.

34. Daw ND, O'Doherty JP, Dayan P, Seymour B, Dolan RJ (2006) Cortical substrates for exploratory decisions in humans. Nature 441: 876–879. Available: http://www.ncbi.nlm.nih.gov/entrez/query.fcgi?cmd=Retrieve&db=PubMed&list_uids=16778890&dopt=Abstract.

35. De Ruiter MB, Vletman DJ, Goudriaan AE, Oosterlaan J, Sjoerds Z, et al. (2009) Response perseveration and ventral prefrontal sensitivity to reward and punishment in male problem gamblers and smokers. Neuropsychopharmacology 34: 1027–1038. Available: http://www.ncbi.nlm.nih.gov/entrez/query.fcgi?cmd=Retrieve&db=PubMed&list_uids=18830241&dopt=Abstract.

36. Delgado MR, Nystrom LE, Fissell C, Noll DC, Fiez JA (2000) Tracking the hemodynamic responses to reward and punishment in the striatum. J Neurophysiol 84: 3072–3077. Available: http://www.ncbi.nlm.nih.gov/entrez/query.fcgi?cmd=Retrieve&db=PubMed&list_uids=11110834&dopt=Abstract.

37. Dichter GS, Richey JA, Rittenberg AM, Sabatino A, Bodfish JW (2012) Reward circuitry function in autism during face anticipation and outcomes. J Autism Dev Disord 42: 147–160. Available: http://www.ncbi.nlm.nih.gov/entrez/query.fcgi?cmd=Retrieve&db=PubMed&list_uids=22187105&dopt=Abstract. Accessed 10 March 2014.

38. Dillon DG, Holmes AJ, Jahn AL, Bogdan R, Wald LL, et al. (2008) Dissociation of neural regions associated with anticipatory versus consummatory phases of incentive processing. Psychophysiology 45: 36–49. Available: http://www.ncbi.nlm.nih.gov/entrez/query.fcgi?cmd=Retrieve&db=PubMed&list_uids=17850241&dopt=Abstract.

39. Dreher JC, Kohn P, Berman KF (2006) Neural coding of distinct statistical properties of reward information in humans. Cereb Cortex 16: 561–573. Available: http://www.ncbi.nlm.nih.gov/entrez/query.fcgi?cmd=Retrieve&db=PubMed&list_uids=16033924&dopt=Abstract.

40. Duka T, Trick L, Nikolaou K, Gray MA, Kempton MJ, et al. (2011) Unique brain areas associated with abstinence control are damaged in multiply detoxified alcoholics. Biol Psychiatry 70: 545–552. Available: http://www.ncbi.nlm.nih.gov/entrez/query.fcgi?cmd=Retrieve&db=PubMed&list_uids=21612768&dopt=Abstract. Accessed 17 March 2014.

41. Elliott R, Agnew Z, Deakin JFW (2008) Medial orbitofrontal cortex codes relative rather than absolute value of financial rewards in humans. Eur J Neurosci 27: 2213–2218. Available: http://www.ncbi.nlm.nih.gov/entrez/query.fcgi?cmd=Retrieve&db=PubMed&list_uids=18445214&dopt=Abstract.

42. Elliott R, Friston KJ, Dolan RJ (2000) Dissociable neural responses in human reward systems. J Neurosci 20: 6159–6165. Available: http://www.ncbi.nlm.nih.gov/entrez/query.fcgi?cmd=Retrieve&db=PubMed&list_uids=10934265&dopt=Abstract.

43. Elliott R, Newman JL, Longe OA, Deakin JFW (2003) Differential response patterns in the striatum and orbitofrontal cortex to financial reward in humans: A parametric functional magnetic resonance imaging study. J Neurosci 23: 303–307. Available: http://www.ncbi.nlm.nih.gov/entrez/query.fcgi?cmd=Retrieve&db=PubMed&list_uids=12514228 &dopt=Abstract.

44. Elliott R, Newman JL, Longe OA, Deakin JFW (2004) Instrumental responding for rewards is associated with enhanced neuronal response in subcortical reward systems. Neuroimage 21: 984–990. Available: http://www.ncbi.nlm.nih.gov/entrez/query.fcgi?cmd=Retrieve&db=PubMed&list_uids=15006665&dopt=Abstract.

45. Elliott R, Rees G, Dolan RJ (1999) Ventromedial prefrontal cortex mediates guessing. Neuropsychologia 37: 403–411. Available:

http://www.ncbi.nlm.nih.gov/entrez/query.fcgi?cmd=Retrieve&db=PubMed&list_uids=10215087&dopt=Abstract.

46. Engelmann JB, Capra CM, Noussair C, Berns GS (2009) Expert financial advice neurobiologically “offloads” financial decision-making under risk. PLoS One 4: 0. Available: http://www.ncbi.nlm.nih.gov/entrez/query.fcgi?cmd=Retrieve&db=PubMed&list_uids=19308261&dopt=Abstract.

47. Engelmann JB, Tamir D (2009) Individual differences in risk preference predict neural responses during financial decision-making. Brain Res 1290: 28–51. Available: http://www.ncbi.nlm.nih.gov/entrez/query.fcgi?cmd=Retrieve&db=PubMed&list_uids=19576868&dopt=Abstract.

48. Ernst M, Nelson EE, Jazbec S, McClure EB, Monk CS, et al. (2005) Amygdala and nucleus accumbens in responses to receipt and omission of gains in adults and adolescents. Neuroimage 25: 1279–1291. Available: http://www.ncbi.nlm.nih.gov/entrez/query.fcgi?cmd=Retrieve&db=PubMed&list_uids=15850746&dopt=Abstract.

49. Ernst M, Nelson EE, McClure EB, Monk CS, Munson S, et al. (2004) Choice selection and reward anticipation: An fMRI study. Neuropsychologia 42: 1585–1597. Available: http://www.ncbi.nlm.nih.gov/entrez/query.fcgi?cmd=Retrieve&db=PubMed&list_uids=15327927&dopt=Abstract.

50. Ersner-Hershfield H, Wimmer GE, Knutson B (2009) Saving for the future self: Neural measures of future self-continuity predict temporal discounting. Soc Cogn Affect Neurosci 1: 85–92. Available: http://www.ncbi.nlm.nih.gov/entrez/query.fcgi?cmd=Retrieve&db=PubMed&list_uids=19047075&dopt=Abstract.

51. Finger EC, Mitchell DG V, Jones M, Blair RJR (2008) Dissociable roles of medial orbitofrontal cortex in human operant extinction learning. Neuroimage 43: 748–755. Available: http://www.ncbi.nlm.nih.gov/entrez/query.fcgi?cmd=Retrieve&db=PubMed&list_uids=18793731&dopt=Abstract.

52. Fleming SM, Whiteley L, Hulme OJ, Sahani M, Dolan RJ (2010) Effects of category-specific costs on neural systems for perceptual decision-making. J Neurophysiol 103: 3238–3247. Available: http://www.ncbi.nlm.nih.gov/entrez/query.fcgi?cmd=Retrieve&db=PubMed&list_uids=20357071&dopt=Abstract. Accessed 17 March 2014.

53. Frangou S, Kington J, Raymont V, Shergill SS (2008) Examining ventral and dorsal prefrontal function in bipolar disorder: A functional magnetic resonance imaging study. Eur Psychiatry 23: 300–308. Available: http://www.ncbi.nlm.nih.gov/entrez/query.fcgi?cmd=Retrieve&db=PubMed&list_uids=17656073&dopt=Abstract.

54. Freyer T, Valerius G, Kuelz AK, Speck O, Glauche V, et al. (2009) Test-retest reliability of event-related functional MRI in a probabilistic reversal learning task. Psychiatry Res 174: 40–46. Available: http://www.ncbi.nlm.nih.gov/entrez/query.fcgi?cmd=Retrieve&db=PubMed&list_uids=19783412&dopt=Abstract.

55. Fukui H, Murai T, Fukuyama H, Hayashi T, Hanakawa T (2005) Functional activity related to risk anticipation during performance of the Iowa gambling task. Neuroimage 24: 253–259. Available: http://www.ncbi.nlm.nih.gov/entrez/query.fcgi?cmd=Retrieve&db=PubMed&list_uids=15588617&dopt=Abstract.

56. Fukui H, Murai T, Shinozaki J, Aso T, Fukuyama H, et al. (2006) The neural basis of social tactics: An fMRI study. Neuroimage 32: 913–920. Available: http://www.ncbi.nlm.nih.gov/entrez/query.fcgi?cmd=Retrieve&db=PubMed&list_uids=16682235&dopt=Abstract.

57. Galvan A, Hare T, Voss H, Glover GH, Casey BJ (2007) Risk-taking and the adolescent brain: Who is at risk? Dev Sci 10: 8–14. Available: http://www.ncbi.nlm.nih.gov/entrez/query.fcgi?cmd=Retrieve&db=PubMed&list_uids=17286837&dopt=Abstract.

58. Glascher J, Hampton AN, O’Doherty JP (2009) Determining a role for ventromedial prefrontal cortex in encoding action-based value signals during reward-related decision

making. Cereb Cortex 19: 483–495. Available: http://www.ncbi.nlm.nih.gov/entrez/query.fcgi?cmd=Retrieve&db=PubMed&list_uids=18550593&dopt=Abstract.

59. Goldstein RZ, Alia-Klein N, Tomasi D, Zhang L, Cottone LA, et al. (2007) Is decreased prefrontal cortical sensitivity to monetary reward associated with impaired motivation and self-control in cocaine addiction? Am J Psychiatry 164: 43–51. Available: http://www.ncbi.nlm.nih.gov/entrez/query.fcgi?cmd=Retrieve&db=PubMed&list_uids=17202543&dopt=Abstract.

60. Guitart-Masip M, Talmi D, Dolan R (2010) Conditioned associations and economic decision biases. Neuroimage 53: 206–214. Available: http://www.ncbi.nlm.nih.gov/entrez/query.fcgi?cmd=Retrieve&db=PubMed&list_uids=20600994&dopt=Abstract. Accessed 4 March 2014.

61. Hampton AN, Adolphs R, Tyszka MJ, O'Doherty JP (2007) Contributions of the amygdala to reward expectancy and choice signals in human prefrontal cortex. Neuron 55: 545–555. Available: http://www.ncbi.nlm.nih.gov/entrez/query.fcgi?cmd=Retrieve&db=PubMed&list_uids=17698008&dopt=Abstract.

62. Hampton AN, Bossaerts P, O'Doherty JP (2006) The role of the ventromedial prefrontal cortex in abstract state-based inference during decision making in humans. J Neurosci 26: 8360–8367. Available: http://www.ncbi.nlm.nih.gov/entrez/query.fcgi?cmd=Retrieve&db=PubMed&list_uids=16899731&dopt=Abstract.

63. Hampton AN, Bossaerts P, O'Doherty JP (2008) Neural correlates of mentalizing-related computations during strategic interactions in humans. Proc Natl Acad Sci 105: 6741–6746. Available: http://www.ncbi.nlm.nih.gov/entrez/query.fcgi?cmd=Retrieve&db=PubMed&list_uids=18427116&dopt=Abstract.

64. Han S, Huettel SA, Raposo A, Adcock RA, Dobbins IG (2010) Functional significance of striatal responses during episodic decisions: recovery or goal attainment? J Neurosci 30: 4767–4775. Available:

http://www.ncbi.nlm.nih.gov/entrez/query.fcgi?cmd=Retrieve&db=PubMed&list_uids=20357127&dopt=Abstract. Accessed 17 March 2014.

65. Hare T, Camerer CF, Rangel A (2009) Self-control in decision-making involves modulation of the vmPFC valuation system. Science (80-) 324: 646–648. Available: http://www.ncbi.nlm.nih.gov/entrez/query.fcgi?cmd=Retrieve&db=PubMed&list_uids=19407204&dopt=Abstract.

66. Hare T, O'Doherty J, Camerer CF, Schultz W, Rangel A (2008) Dissociating the role of the orbitofrontal cortex and the striatum in the computation of goal values and prediction errors. J Neurosci 28: 5623–5630. Available: http://www.ncbi.nlm.nih.gov/entrez/query.fcgi?cmd=Retrieve&db=PubMed&list_uids=18509023&dopt=Abstract.

67. Hare TA, Camerer CF, Knoepfle DT, Rangel A (2010) Value computations in ventral medial prefrontal cortex during charitable decision making incorporate input from regions involved in social cognition. J Neurosci 30: 583–590. Available: http://www.ncbi.nlm.nih.gov/entrez/query.fcgi?cmd=Retrieve&db=PubMed&list_uids=20071521&dopt=Abstract. Accessed 23 January 2014.

68. Hartstra E, Oldenburg JFE, Van Leijenhorst L, Rombouts SARB, Crone EA (2010) Brain regions involved in the learning and application of reward rules in a two-deck gambling task. Neuropsychologia 48: 1438–1446. Available: http://www.ncbi.nlm.nih.gov/entrez/query.fcgi?cmd=Retrieve&db=PubMed&list_uids=20105435&dopt=Abstract. Accessed 10 March 2014.

69. Haruno M, Kuroda T, Doya K, Toyama K, Kimura M, et al. (2004) A neural correlate of reward-based behavioral learning in caudate nucleus: A functional magnetic resonance imaging study of a stochastic decision task. J Neurosci 24: 1660–1665. Available: http://www.ncbi.nlm.nih.gov/entrez/query.fcgi?cmd=Retrieve&db=PubMed&list_uids=14973239&dopt=Abstract.

70. Hsu M, Bhatt M, Adolphs R, Tranel D, Camerer CF (2005) Neural systems responding to degrees of uncertainty in human decision-making. Science (80-) 310: 1680–1683. Available: http://www.ncbi.nlm.nih.gov/entrez/query.fcgi?cmd=Retrieve&db=PubMed&list_uids=16339445&dopt=Abstract.

71. Hsu M, Krajbich I, Zhao C, Camerer CF (2009) Neural response to reward anticipation under risk is nonlinear in probabilities. J Neurosci 29: 2231–2237. Available: http://www.ncbi.nlm.nih.gov/entrez/query.fcgi?cmd=Retrieve&db=PubMed&list_uids=19228976&dopt=Abstract.

72. Huettel SA (2006) Behavioral, but not reward, risk modulates activation of prefrontal, parietal, and insular cortices. Cogn Affect Behav Neurosci 2: 141–151. Available: http://www.ncbi.nlm.nih.gov/entrez/query.fcgi?cmd=Retrieve&db=PubMed&list_uids=17007234&dopt=Abstract.

73. Huettel SA, Stowe CJ, Gordon EM, Warner BT, Platt ML (2006) Neural signatures of economic preferences for risk and ambiguity. Neuron 49: 765–775. Available: http://www.ncbi.nlm.nih.gov/entrez/query.fcgi?cmd=Retrieve&db=PubMed&list_uids=16504951&dopt=Abstract.

74. Izuma K, Saito DN, Sadato N (2008) Processing of social and monetary rewards in the human striatum. Neuron 58: 284–294. Available: http://www.ncbi.nlm.nih.gov/entrez/query.fcgi?cmd=Retrieve&db=PubMed&list_uids=18439412&dopt=Abstract.

75. Jocham G, Neumann J, Klein TA, Danielmeier C, Ullsperger M (2009) Adaptive coding of action values in the human rostral cingulate zone. J Neurosci 29: 7489–7496. Available: http://www.ncbi.nlm.nih.gov/entrez/query.fcgi?cmd=Retrieve&db=PubMed&list_uids=19515916&dopt=Abstract.

76. Juckel G, Schlagenhauf F, Koslowski M, Wustenberg T, Villringer A, et al. (2006) Dysfunction of ventral striatal reward prediction in schizophrenia. Neuroimage 29: 409–416. Available: http://www.ncbi.nlm.nih.gov/entrez/query.fcgi?cmd=Retrieve&db=PubMed&list_uids=16139525&dopt=Abstract.

77. Kahnt T, Heinzle J, Park SQ, Haynes J-D (2011) Decoding different roles for vmPFC and dlPFC in multi-attribute decision making. Neuroimage 56: 709–715. Available: http://www.ncbi.nlm.nih.gov/entrez/query.fcgi?cmd=Retrieve&db=PubMed&list_uids=20510371&dopt=Abstract. Accessed 10 March 2014.

78. Kahnt T, Heinzle J, Park SQ, Haynes J-D (2010) The neural code of reward anticipation in human orbitofrontal cortex. Proc Natl Acad Sci U S A 107: 6010–6015. Available: http://www.ncbi.nlm.nih.gov/entrez/query.fcgi?cmd=Retrieve&db=PubMed&list_uids=20231475&dopt=Abstract. Accessed 17 March 2014.

79. Kim H, Shimojo S, O'Doherty JP (2006) Is avoiding an aversive outcome rewarding? Neural substrates of avoidance learning in the human brain. PLoS Biol 4: 1453–1461. Available: http://www.ncbi.nlm.nih.gov/entrez/query.fcgi?cmd=Retrieve&db=PubMed&list_uids=16802856&dopt=Abstract.

80. Kirsch P, Reuter M, Mier D, Lonsdorf T, Strark R, et al. (2006) Imaging gene-substance interactions: The effect of the DRD2 TaqIA polymorphism and the dopamine agonist bromocriptine on the brain activation during the anticipation of reward. Neurosci Lett 405: 196–201. Available: http://www.ncbi.nlm.nih.gov/entrez/query.fcgi?cmd=Retrieve&db=PubMed&list_uids=16901644&dopt=Abstract.

81. Kirsch P, Schienle A, Stark R, Sammer G, Blecker C, et al. (2003) Anticipation of reward in a nonaversive differential conditioning paradigm and the brain reward system: An event-related fMRI study. Neuroimage 20: 1086–1095. Available: http://www.ncbi.nlm.nih.gov/entrez/query.fcgi?cmd=Retrieve&db=PubMed&list_uids=14568478&dopt=Abstract.

82. Knutson B, Adams CM, Fong GW, Hommer D (2001) Anticipation of increasing monetary reward selectively recruits nucleus accumbens. J Neurosci 21: 1–5. Available: http://www.ncbi.nlm.nih.gov/entrez/query.fcgi?cmd=Retrieve&db=PubMed&list_uids=11459880 &dopt=Abstract.

83. Knutson B, Bhanji JP, Cooney RE, Atlas LY, Gotlib IH (2008) Neural responses to monetary incentives in major depression. Biol Psychiatry 63: 686–692. Available: http://www.ncbi.nlm.nih.gov/entrez/query.fcgi?cmd=Retrieve&db=PubMed&list_uids=17916330&dopt=Abstract.

84. Knutson B, Bjork JM, Fong GW, Hommer D, Mattay VS, et al. (2004) Amphetamine modulates human incentive processing. Neuron 43: 261–269. Available:

http://www.ncbi.nlm.nih.gov/entrez/query.fcgi?cmd=Retrieve&db=PubMed&list_uids=15260961&dopt=Abstract.

85. Knutson B, Fong GW, Adams CM, Varner JL, Hommer D (2001) Dissociation of reward anticipation and outcome with event-related fMRI. Neuroreport 12: 3683–3687. Available: http://www.ncbi.nlm.nih.gov/entrez/query.fcgi?cmd=Retrieve&db=PubMed&list_uids=11726774&dopt=Abstract.

86. Knutson B, Fong GW, Bennett SM, Adams CM, Hommer D (2003) A region of mesial prefrontal cortex tracks monetarily rewarding outcomes: Characterization with rapid event-related fMRI. Neuroimage 18: 263–272. Available: http://www.ncbi.nlm.nih.gov/entrez/query.fcgi?cmd=Retrieve&db=PubMed&list_uids=12595181&dopt=Abstract.

87. Knutson B, Rick S, Wimmer GE, Prelec D, Loewenstein G (2007) Neural predictors of purchases. Neuron 53: 147–156. Available: http://www.ncbi.nlm.nih.gov/entrez/query.fcgi?cmd=Retrieve&db=PubMed&list_uids=17196537&dopt=Abstract.

88. Knutson B, Taylor J, Kaufman M, Peterson R, Glover GH (2005) Distributed neural representation of expected value. J Neurosci 25: 4806–4812. Available: http://www.ncbi.nlm.nih.gov/entrez/query.fcgi?cmd=Retrieve&db=PubMed&list_uids=15888656&dopt=Abstract.

89. Knutson B, Westdorp A, Kaiser E, Hommer D (2000) FMRI visualization of brain activity during a monetary incentive delay task. Neuroimage 12: 20–27. Available: http://www.ncbi.nlm.nih.gov/entrez/query.fcgi?cmd=Retrieve&db=PubMed&list_uids=10875899&dopt=Abstract.

90. Knutson B, Wimmer GE, Kuhnen CM, Winkielman P (2008) Nucleus accumbens activation mediates the influence of reward cues on financial risk taking. Neuroreport 19: 509–513. Available: http://www.ncbi.nlm.nih.gov/entrez/query.fcgi?cmd=Retrieve&db=PubMed&list_uids=18388729&dopt=Abstract.

91. Knutson B, Wimmer GE, Rick S, Hollon NG, Prelec D, et al. (2008) Neural antecedents of the endowment effect. Neuron 58: 814–822. Available: http://www.ncbi.nlm.nih.gov/entrez/query.fcgi?cmd=Retrieve&db=PubMed&list_uids=18549791&dopt=Abstract.

92. Koeneke S, Pedroni AF, Dieckmann A, Bosch V, Jancke L (2008) Individual preferences modulate incentive values: Evidence from functional MRI. Behav Brain Funct 4: 1–13. Available: http://www.ncbi.nlm.nih.gov/entrez/query.fcgi?cmd=Retrieve&db=PubMed&list_uids=19032746&dopt=Abstract.

93. Kramer UM, Jansma H, Tempelmann C, Munte TF (2007) Tit-for-tat: The neural basis of reactive aggression. Neuroimage 38: 203–211. Available: http://www.ncbi.nlm.nih.gov/entrez/query.fcgi?cmd=Retrieve&db=PubMed&list_uids=17765572&dopt=Abstract.

94. Kuhnen CM, Knutson B (2005) The neural basis of financial risk taking. Neuron 47: 763–770. Available: http://www.ncbi.nlm.nih.gov/entrez/query.fcgi?cmd=Retrieve&db=PubMed&list_uids=16129404&dopt=Abstract.

95. Labudda K, Woermann FG, Mertens M, Pohlmann-Eden B, Markowitsch HJ, et al. (2008) Neural correlates of decision making with explicit information about probabilities and incentives in elderly healthy subjects. Exp Brain Res 187: 641–650. Available: http://www.ncbi.nlm.nih.gov/entrez/query.fcgi?cmd=Retrieve&db=PubMed&list_uids=18320179&dopt=Abstract.

96. Lawrence NS, Jollant F, O'Daly O, Zelaya FO, Phillips ML (2009) Distinct roles of prefrontal cortical subregions in the Iowa Gambling Task. Cereb Cortex 19: 1134–1143. Available: http://www.ncbi.nlm.nih.gov/entrez/query.fcgi?cmd=Retrieve&db=PubMed&list_uids=18787233&dopt=Abstract.

97. Lee TMC, Leung AW, Fox PT, Gao JH, Chan CCH (2008) Age-related differences in neural activities during risk taking as revealed by functional MRI. Soc Cogn Affect Neurosci 3: 7–15. Available:

http://www.ncbi.nlm.nih.gov/entrez/query.fcgi?cmd=Retrieve&db=PubMed&list_uids=19015090&dopt=Abstract.

98. Linke J, Kirsch P, King A V, Gass A, Hennerici MG, et al. (2010) Motivational orientation modulates the neural response to reward. Neuroimage 49: 2618–2625. Available: http://www.ncbi.nlm.nih.gov/entrez/query.fcgi?cmd=Retrieve&db=PubMed&list_uids=19770058&dopt=Abstract. Accessed 10 March 2014.

99. Little DM, Shin SS, Sisco SM, Thulborn KR (2006) Event-related fMRI of category learning: Differences in classification and feedback networks. Brain Cogn 60: 244–252. Available: http://www.ncbi.nlm.nih.gov/entrez/query.fcgi?cmd=Retrieve&db=PubMed&list_uids=16426719&dopt=Abstract.

100. Liu X, Powell DK, Wang H, Gold BT, Corbly CR, et al. (2007) Functional dissociation in frontal and striatal areas for processing of positive and negative reward information. J Neurosci 27: 4587–4597. Available: http://www.ncbi.nlm.nih.gov/entrez/query.fcgi?cmd=Retrieve&db=PubMed&list_uids=17460071&dopt=Abstract.

101. Marco-Pallares J, Muller S V, Munte TF (2007) Learning by doing: An fMRI study of feedback-related brain activations. Neuroreport 18: 1423–1426. Available: http://www.ncbi.nlm.nih.gov/entrez/query.fcgi?cmd=Retrieve&db=PubMed&list_uids=17712267&dopt=Abstract.

102. Marsh AA, Blair KS, Vythilingam M, Busis S, Blair RJR (2007) Response options and expectations of reward in decision-making: The differential roles of dorsal and rostral anterior cingulate cortex. Neuroimage 35: 979–988. Available: http://www.ncbi.nlm.nih.gov/entrez/query.fcgi?cmd=Retrieve&db=PubMed&list_uids=17292631&dopt=Abstract.

103. Martin-Soelch C, Chevalley AF, G K u00fcnig, Missimer J, Magyar S, et al. (2001) Changes in reward-induced brain activation in opiate addicts. Eur J Neurosci 14: 1360–1368. Available: http://www.ncbi.nlm.nih.gov/entrez/query.fcgi?cmd=Retrieve&db=PubMed&list_uids=11703464&dopt=Abstract.

104. Martin-Soelch C, Missimer J, Leenders KL, Schultz W (2003) Neural activity related to the processing of increasing monetary reward in smokers and nonsmokers. Eur J Neurosci 18: 680–688. Available: http://www.ncbi.nlm.nih.gov/entrez/query.fcgi?cmd=Retrieve&db=PubMed&list_uids=12911764&dopt=Abstract.

105. Martin-Solch C, Magyar S, G K u00fcnig, Missimer J, Schultz W, et al. (2001) Changes in brain activation associated with reward processing in smokers and nonsmokers: A positron emission tomography study. Exp Brain Res 139: 278–286. Available: http://www.ncbi.nlm.nih.gov/entrez/query.fcgi?cmd=Retrieve&db=PubMed&list_uids=11545466&dopt=Abstract.

106. Matthews SC, Simmons AN, Lane SD, Paulus MP (2004) Selective activation of the nucleus accumbens during risk-taking decision making. Neuroreport 15: 2123–2127. Available: http://www.ncbi.nlm.nih.gov/entrez/query.fcgi?cmd=Retrieve&db=PubMed&list_uids=15486494&dopt=Abstract.

107. McClure SM, Berns GS, Montague PR (2003) Temporal predection errors in a passive learning task activate human striatum. Neuron 38: 339–346. Available: http://www.ncbi.nlm.nih.gov/entrez/query.fcgi?cmd=Retrieve&db=PubMed&list_uids=12718866&dopt=Abstract.

108. Miedl SF, Fehr T, Meyer G, Herrmann M (2010) Neurobiological correlates of problem gambling in a quasi-realistic blackjack scenario as revealed by fMRI. Psychiatry Res 181: 165–173. Available: http://www.ncbi.nlm.nih.gov/entrez/query.fcgi?cmd=Retrieve&db=PubMed&list_uids=20138482&dopt=Abstract. Accessed 16 March 2014.

109. Mitchell DG V, Luo Q, Avny SB, Kasprzycki T, Gupta K, et al. (2009) Adapting to dynamic stimulus-response values: Differential contributions of inferior frontal, dorsomedial, and dorsolateral regions of prefrontal cortex to decision making. J Neurosci 29: 10827–10834. Available: http://www.ncbi.nlm.nih.gov/entrez/query.fcgi?cmd=Retrieve&db=PubMed&list_uids=19726640&dopt=Abstract.

110. Mitchell DG V, Rhodes RA, Pine DS, Blair RJ (2008) The contribution of ventrolateral and dorsolateral prefrontal cortex to response reversal. Behav Brain Res 187: 80–87. Available: http://www.ncbi.nlm.nih.gov/entrez/query.fcgi?cmd=Retrieve&db=PubMed&list_uids=17950474&dopt=Abstract.

111. Mobbs D, Petrovic P, Marchant JL, Hassabis D, Weiskopf N, et al. (2007) When fear is near: Threat imminence elicits prefrontal-periaqueductal gray shifts in humans. Science (80-) 317: 1079–1083. Available: http://www.ncbi.nlm.nih.gov/entrez/query.fcgi?cmd=Retrieve&db=PubMed&list_uids=17717184&dopt=Abstract.

112. Nieuwenhuis S, Heslenfeld DJ, Alting von Geusau NJ, Mars RB, Holroyd CB, et al. (2005) Activity in human reward-sensitive brain areas is strongly context dependent. Neuroimage 25: 1302–1309. Available: http://www.ncbi.nlm.nih.gov/entrez/query.fcgi?cmd=Retrieve&db=PubMed&list_uids=15945130&dopt=Abstract.

113. Nieuwenhuis S, Slagter HA, Alting von Geusau NJ, Heslenfeld DJ, Holroyd CB (2005) Knowing good from bad: Differential activation of human cortical areas by positive and negative outcomes. Eur J Neurosci 21: 3161–3168. Available: http://www.ncbi.nlm.nih.gov/entrez/query.fcgi?cmd=Retrieve&db=PubMed&list_uids=15978024&dopt=Abstract.

114. O'Doherty J, Dayan P, Schultz J, Deichmann R, Friston KJ, et al. (2004) Dissociable roles of ventral and dorsal striatum in instrumental conditioning. Science (80-) 304: 452–454. Available: http://www.ncbi.nlm.nih.gov/entrez/query.fcgi?cmd=Retrieve&db=PubMed&list_uids=15087550&dopt=Abstract.

115. O'Doherty J, Kringelbach ML, Rolls ET, Hornak J, Andrews C (2001) Abstract reward and punishment representations in the human orbitofrontal cortex. Nat Neurosci 4: 95–102. Available: http://www.ncbi.nlm.nih.gov/entrez/query.fcgi?cmd=Retrieve&db=PubMed&list_uids=11135651&dopt=Abstract.

116. Palminteri S, Boraud T, Lafargue G, Dubois B, Pessiglione M (2009) Brain hemispheres selectively track the expected value of contralateral options. J Neurosci 43: 13465–13472. Available: http://www.ncbi.nlm.nih.gov/entrez/query.fcgi?cmd=Retrieve&db=PubMed&list_uids=19864559&dopt=Abstract.

117. Park SQ, Kahnt T, Beck A, Cohen MX, Dolan RJ, et al. (2010) Prefrontal cortex fails to learn from reward prediction errors in alcohol dependence. J Neurosci 30: 7749–7753. Available: http://www.ncbi.nlm.nih.gov/entrez/query.fcgi?cmd=Retrieve&db=PubMed&list_uids=20519550&dopt=Abstract. Accessed 17 March 2014.

118. Paulus MP, Hozack N, Zauscher B, McDowell JE, Frank L, et al. (2001) Prefrontal, parietal, and temporal cortex networks underlie decision-making in the presence of uncertainty. Neuroimage 13: 91–100. Available: http://www.ncbi.nlm.nih.gov/entrez/query.fcgi?cmd=Retrieve&db=PubMed&list_uids=11133312&dopt=Abstract.

119. Paulus MP, Rogalsky C, Simmons A, Feinstein JS, Stein MB (2003) Increased activation in the right insula during risk-taking decision making is related to harm avoidance and neuroticism. Neuroimage 4: 1439–1448. Available: http://www.ncbi.nlm.nih.gov/entrez/query.fcgi?cmd=Retrieve&db=PubMed&list_uids=12948701&dopt=Abstract.

120. Pessiglione M, Seymour B, Flandin G, Dolan RJ, Frith CD (2006) Dopamine-dependent prediction errors underpin reward-seeking behaviour in humans. Nature 442: 1042–1045. Available: http://www.ncbi.nlm.nih.gov/entrez/query.fcgi?cmd=Retrieve&db=PubMed&list_uids=16929307&dopt=Abstract.

121. Plassmann H, O’Doherty J, Rangel A (2007) Orbitofrontal cortex encodes willingness to pay in everyday economic transactions. J Neurosci 27: 9984–9988. Available: http://www.ncbi.nlm.nih.gov/entrez/query.fcgi?cmd=Retrieve&db=PubMed&list_uids=17855612&dopt=Abstract.

122. Pochon JB, Levy R, Fossati P, Lehericy S, Poline JB, et al. (2002) The neural system that bridges reward and cognition in humans: An fMRI study. Proc Natl Acad Sci 99: 5669–5674. Available: http://www.ncbi.nlm.nih.gov/entrez/query.fcgi?cmd=Retrieve&db=PubMed&list_uids=11960021&dopt=Abstract.

123. Preuschoff K, Bossaerts P, Quartz SR (2006) Neural differentiation of expected reward and risk in human subcortical structures. Neuron 51: 381–390. Available: http://www.ncbi.nlm.nih.gov/entrez/query.fcgi?cmd=Retrieve&db=PubMed&list_uids=16880132&dopt=Abstract.

124. Preuschoff K, Quartz SR, Bossaerts P (2008) Human insula activation reflects risk prediction errors as well as risk. J Neurosci 28: 2745–2752. Available: http://www.ncbi.nlm.nih.gov/entrez/query.fcgi?cmd=Retrieve&db=PubMed&list_uids=18337404&dopt=Abstract.

125. Ramnani N, Elliott R, Athwal BS, Passingham RE (2004) Prediction error for free monetary reward in the human prefrontal cortex. Neuroimage 23: 777–786. Available: http://www.ncbi.nlm.nih.gov/entrez/query.fcgi?cmd=Retrieve&db=PubMed&list_uids=15528079&dopt=Abstract.

126. Ramnani N, Miall RC (2003) Instructed delay activity in the human prefrontal cortex is modulated by monetary reward expectation. Cereb Cortex 13: 318–327. Available: http://www.ncbi.nlm.nih.gov/entrez/query.fcgi?cmd=Retrieve&db=PubMed&list_uids=12571121&dopt=Abstract.

127. Rao H, Korczykowski M, Pluta J, Hoang A, Detre JA (2008) Neural correlates of voluntary and involuntary risk taking in the human brain: An fMRI Study of the Balloon Analog Risk Task (BART). Neuroimage 42: 902–910. Available: http://www.ncbi.nlm.nih.gov/entrez/query.fcgi?cmd=Retrieve&db=PubMed&list_uids=18582578&dopt=Abstract.

128. Remijnse PL, Nielen MMA, Uylings HBM, Veltman DJ (2005) Neural correlates of a reversal learning task with an affectively neutral baseline: An event-related fMRI study. Neuroimage 26: 609–618. Available:

http://www.ncbi.nlm.nih.gov/entrez/query.fcgi?cmd=Retrieve&db=PubMed&list_uids=15907318&dopt=Abstract.

129. Remijnse PL, Nielen MMA, van Balkom AJLM, Cath DC, van Oppen P, et al. (2006) Reduced orbitofrontal-striatal activity on a reversal learning task in obsessive-compulsive disorder. Arch Gen Psychiatry 63: 1225–1236. Available: http://www.ncbi.nlm.nih.gov/entrez/query.fcgi?cmd=Retrieve&db=PubMed&list_uids=17088503&dopt=Abstract.

130. Remijnse PL, Nielen MMA, van Balkom AJLM, Hendriks GJ, Hoogendijk WJ, et al. (2009) Differential frontal-striatal and paralimbic activity during reversal learning in major depressive disorder and obsessive-compulsive disorder. Psychol Med 39: 1503–1518. Available: http://www.ncbi.nlm.nih.gov/entrez/query.fcgi?cmd=Retrieve&db=PubMed&list_uids=19171077&dopt=Abstract.

131. Rilling JK, Sanfey AG, Aronson JA, Nystrom LE, Cohen JD (2004) Opposing BOLD responses to reciprocated and unreciprocated altruism in putative reward pathways. Neuroreport 15: 2539–2543. Available: http://www.ncbi.nlm.nih.gov/entrez/query.fcgi?cmd=Retrieve&db=PubMed&list_uids=15538191&dopt=Abstract.

132. Rogers RD, Owen AM, Middleton HC, Williams EJ, Pickard JD, et al. (1999) Choosing between small, likely rewards and large, unlikely rewards activates inferior and orbital prefrontal cortex. J Neurosci 19: 9029–9038. Available: http://www.ncbi.nlm.nih.gov/entrez/query.fcgi?cmd=Retrieve&db=PubMed&list_uids=10516320&dopt=Abstract.

133. Sailer U, Robinson S, Fischmeister FPS, Moser E, Krypsin-Exner I, et al. (2007) Imaging the changing role of feedback during learning in decision-making. Neuroimage 37: 1474–1486. Available: http://www.ncbi.nlm.nih.gov/entrez/query.fcgi?cmd=Retrieve&db=PubMed&list_uids=17698371&dopt=Abstract.

134. Samanez-Larkin GR, Gibbs SEB, Khanna K, Nielsen L, Carstensen LL, et al. (2007) Anticipation of monetary gain but not loss in healthy older adults. Nat Neurosci 10: 787–791.

Available:
http://www.ncbi.nlm.nih.gov/entrez/query.fcgi?cmd=Retrieve&db=PubMed&list_uids=17468751&dopt=Abstract.

135. Samanez-Larkin GR, Hollon NG, Carstensen LL, Knutson B (2008) Individual differences in insular sensitivity during loss anticipation predict avoidance learning. Psychol Sci 19: 320–323. Available: http://www.ncbi.nlm.nih.gov/entrez/query.fcgi?cmd=Retrieve&db=PubMed&list_uids=18399882&dopt=Abstract.

136. Sanfey AG, Rilling JK, Aronson JA, Nystrom LE, Cohen JD (2003) The neural basis of economic decision-making in the ultimatum game. Science (80-) 300: 1755–1758. Available: http://www.ncbi.nlm.nih.gov/entrez/query.fcgi?cmd=Retrieve&db=PubMed&list_uids=12805551&dopt=Abstract.

137. Schaefer M, Rotte M (2007) Thinking on luxury or pragmatic brand products: Brain responses to different categories of culturally based brands. Brain Res 1165: 98–104. Available: http://www.ncbi.nlm.nih.gov/entrez/query.fcgi?cmd=Retrieve&db=PubMed&list_uids=17655834&dopt=Abstract.

138. Scheres A, Milham MP, Knutson B, Castellanos FX (2007) Ventral striatal hyporesponsiveness during reward anticipation in attention-deficit/hyperactivity disorder. Biol Psychiatry 61: 720–724. Available: http://www.ncbi.nlm.nih.gov/entrez/query.fcgi?cmd=Retrieve&db=PubMed&list_uids=16950228&dopt=Abstract.

139. Schlagenhauf F, Juckel G, Koslowski M, Kahnt T, Knutson B, et al. (2008) Reward system activation in schizophrenic patients switched from typical neuroleptics to olanzapine. Psychopharmacology (Berl) 196: 673–684. Available: http://www.ncbi.nlm.nih.gov/entrez/query.fcgi?cmd=Retrieve&db=PubMed&list_uids=18097655&dopt=Abstract.

140. Schonberg T, Daw ND, Joel D, O'Doherty JP (2007) Reinforcement learning signals in the human striatum distinguish learners from nonlearners during reward-based decision making.

J Neurosci 27: 12860–12867. Available: http://www.ncbi.nlm.nih.gov/entrez/query.fcgi?cmd=Retrieve&db=PubMed&list_uids=18032658&dopt=Abstract.

141. Seymour B, Daw N, Dayan P, Singer T, Dolan R (2007) Differential encoding of losses and gains in the human striatum. J Neurosci 27: 4826–4831. Available: http://www.ncbi.nlm.nih.gov/entrez/query.fcgi?cmd=Retrieve&db=PubMed&list_uids=17475790&dopt=Abstract.

142. Simões-Franklin C, Hester R, Shpaner M, Foxe JJ, Garavan H (2010) Executive function and error detection: The effect of motivation on cingulate and ventral striatum activity. Hum Brain Mapp 31: 458–469. Available: http://www.ncbi.nlm.nih.gov/entrez/query.fcgi?cmd=Retrieve&db=PubMed&list_uids=19718655&dopt=Abstract. Accessed 17 March 2014.

143. Smith BW, Mitchell DG V, Hardin MG, Jazbec S, Fridberg D, et al. (2009) Neural substrates of reward magnitude, probability, and risk during a wheel of fortune decision-making task. Neuroimage 44: 600–609. Available: http://www.ncbi.nlm.nih.gov/entrez/query.fcgi?cmd=Retrieve&db=PubMed&list_uids=18804540&dopt=Abstract.

144. Smith D V, Hayden BY, Truong T-K, Song AW, Platt ML, et al. (2010) Distinct value signals in anterior and posterior ventromedial prefrontal cortex. J Neurosci 30: 2490–2495. Available: http://www.ncbi.nlm.nih.gov/entrez/query.fcgi?cmd=Retrieve&db=PubMed&list_uids=20164333&dopt=Abstract. Accessed 17 March 2014.

145. Smoski M, Felder J, Bizzell J, Green SR, Ernst M, et al. (2009) fMRI of alterations in reward selection, anticipation, and feedback in major depressive disorder. J Affect Disord 118: 69–78. Available: http://www.ncbi.nlm.nih.gov/entrez/query.fcgi?cmd=Retrieve&db=PubMed&list_uids=19261334&dopt=Abstract.

146. Spreckelmeyer KN, Krach S, Kohls G, Rademacher L, Irmak A, et al. (2009) Anticipation of monetary and social reward differently activates mesolimbic brain structures in men and women. Soc Cogn Affect Neurosci 4: 158–165. Available:

http://www.ncbi.nlm.nih.gov/entrez/query.fcgi?cmd=Retrieve&db=PubMed&list_uids=19174537&dopt=Abstract.

147. Sripada CS, Angstadt M, Banks S, Nathan PJ, Liberzon I, et al. (2009) Functional neuroimaging of mentalizing during the trust game in social anxiety disorder. Neuroreport 20: 984–989. Available: http://www.ncbi.nlm.nih.gov/entrez/query.fcgi?cmd=Retrieve&db=PubMed&list_uids=19521264&dopt=Abstract.

148. Steele JD, Meyer ME, Ebmeier KP (2004) Neural predictive error signal correlates with depressive illness severity in a game paradigm. Neuroimage 23: 269–280. Available: http://www.ncbi.nlm.nih.gov/entrez/query.fcgi?cmd=Retrieve&db=PubMed&list_uids=15325374&dopt=Abstract.

149. Strohle A, Stoy M, Wrase J, Schwarzer S, Schlagenhauf F, et al. (2008) Reward anticipation and outcomes in adult males with attention-deficit/hyperactivity disorder. Neuroimage 39: 966–972. Available: http://www.ncbi.nlm.nih.gov/entrez/query.fcgi?cmd=Retrieve&db=PubMed&list_uids=17996464&dopt=Abstract.

150. Tanaka SC, Balleine BW, O'Doherty JP (2008) Calculating consequences: Brain systems that encode the causal effects of actions. J Neurosci 28: 6750–6755. Available: http://www.ncbi.nlm.nih.gov/entrez/query.fcgi?cmd=Retrieve&db=PubMed&list_uids=18579749&dopt=Abstract.

151. Tobler PN, Christopoulos GI, O'Doherty JP, Dolan RJ, Schultz W (2008) Neuronal distortions of reward probability without choice. J Neurosci 28: 11703–11711. Available: http://www.ncbi.nlm.nih.gov/entrez/query.fcgi?cmd=Retrieve&db=PubMed&list_uids=18987206&dopt=Abstract.

152. Tobler PN, O'Doherty JP, Dolan RJ, Schultz W (2007) Reward value coding distinct from risk attitude-related uncertainty coding in human reward systems. J Neurophysiol 97: 1621–1632. Available: http://www.ncbi.nlm.nih.gov/entrez/query.fcgi?cmd=Retrieve&db=PubMed&list_uids=17122317&dopt=Abstract.

153. Tricomi E, Balleine B, O'Doherty JP (2009) A specific role for posterior dorsolateral striatum in human habit learning. Eur J Neurosci 29: 2225–2232. Available: http://www.ncbi.nlm.nih.gov/entrez/query.fcgi?cmd=Retrieve&db=PubMed&list_uids=19490086&dopt=Abstract.

154. Ullsperger M, Yves von Cramon D (2003) Error monitoring using external feedback: Specific roles of the habenular complex, the reward system, and the cingulate motor area revealed by the functional magnetic resonance imaging. J Neurosci 23: 4308–4314. Available: http://www.ncbi.nlm.nih.gov/entrez/query.fcgi?cmd=Retrieve&db=PubMed&list_uids=12764119 &dopt=Abstract.

155. Valentin V V, O'Doherty JP (2009) Overlapping prediction errors in dorsal striatum during instrumental learning with juice and money reward in the human brain. J Neurophysiol 102: 3384–3391. Available: http://www.ncbi.nlm.nih.gov/entrez/query.fcgi?cmd=Retrieve&db=PubMed&list_uids=19793875&dopt=Abstract.

156. Van Leijenhorst L, Crone EA, Bunge SA (2006) Neural correlates of developmental differences in risk estimation and feedback processing. Neuropsychologia 44: 2158–2170. Available: http://www.ncbi.nlm.nih.gov/entrez/query.fcgi?cmd=Retrieve&db=PubMed&list_uids=16574168&dopt=Abstract.

157. Venkatraman V, Payne JW, Bettman JR, Luce MF, Huettel SA (2009) Separate neural mechanisms underlie choices and strategic preferences in risky decision making. Neuron 62: 593–602. Available: http://www.ncbi.nlm.nih.gov/entrez/query.fcgi?cmd=Retrieve&db=PubMed&list_uids=19477159&dopt=Abstract.

158. Venkatraman V, Huettel SA, Chuah LYM, Payne JW, Chee MWL (2011) Sleep deprivation biases the neural mechanisms underlying economic preferences. J Neurosci 31: 3712–3718. Available: http://www.ncbi.nlm.nih.gov/entrez/query.fcgi?cmd=Retrieve&db=PubMed&list_uids=21389226&dopt=Abstract. Accessed 17 March 2014.

159. Volz KG, Schubotz RI, Yves von Cramon D (2003) Predicting events of varying probability: Uncertainty investigated by fMRI. Neuroimage 19: 271–280. Available: http://www.ncbi.nlm.nih.gov/entrez/query.fcgi?cmd=Retrieve&db=PubMed&list_uids=12814578&dopt=Abstract.

160. Volz KG, Schubotz RI, Yves von Cramon D (2004) Why am I unsure? Internal and external attributions of uncertainty dissociated by fMRI. Neuroimage 21: 848–857. Available: http://www.ncbi.nlm.nih.gov/entrez/query.fcgi?cmd=Retrieve&db=PubMed&list_uids=15006651&dopt=Abstract.

161. Weber BJ, Huettel SA (2008) The neural substrates of probabilistic and intertemporal decision making. Brain Res 1234: 104–115. Available: http://www.ncbi.nlm.nih.gov/entrez/query.fcgi?cmd=Retrieve&db=PubMed&list_uids=18710652&dopt=Abstract.

162. Weber B, Rangel A, Wibral M, Falk A (2009) The medial prefrontal cortex exhibits money illusion. Proc Natl Acad Sci 13: 5025–5028. Available: http://www.ncbi.nlm.nih.gov/entrez/query.fcgi?cmd=Retrieve&db=PubMed&list_uids=19307555&dopt=Abstract.

163. Wrase J, Kahnt T, Schlagenhauf F, Beck A, Cohen MX, et al. (2007) Different neural systems adjust motor behavior in response to reward and punishment. Neuroimage 36: 1253–1262. Available: http://www.ncbi.nlm.nih.gov/entrez/query.fcgi?cmd=Retrieve&db=PubMed&list_uids=17521924&dopt=Abstract.

164. Wrase J, Schlagenhauf F, Kienast T, Wustenberg T, Bermpohl F, et al. (2007) Dysfunction of reward processing correlates with alcohol craving in detoxified alcoholics. Neuroimage 35: 787–794. Available: http://www.ncbi.nlm.nih.gov/entrez/query.fcgi?cmd=Retrieve&db=PubMed&list_uids=17291784&dopt=Abstract.

165. Wunderlich K, Rangel A, O'Doherty JP (2009) Neural computations underlying action-based decisionmaking in the human brain. Proc Natl Acad Sci 106: 17199–17204. Available: http://www.ncbi.nlm.nih.gov/entrez/query.fcgi?cmd=Retrieve&db=PubMed&list_uids=19805082&dopt=Abstract.

166. Xue G, Lu ZL, Levin IP, Weller JA, Li X, et al. (2009) Functional dissociations of risk and reward processing in the medial prefrontal cortex. Cereb Cortex 5: 1019–1027. Available: http://www.ncbi.nlm.nih.gov/entrez/query.fcgi?cmd=Retrieve&db=PubMed&list_uids=18842669&dopt=Abstract.

167. Yacubian J, Glascher J, Schroeder K, Sommer T, Braus DF, et al. (2006) Dissociable systems for gain- and loss-related value predictions and errors of prediction in the human brain. J Neurosci 26: 9530–9537. Available: http://www.ncbi.nlm.nih.gov/entrez/query.fcgi?cmd=Retrieve&db=PubMed&list_uids=16971537&dopt=Abstract.

168. Zheng H, Wang XT, Zhu L (2010) Framing effects: behavioral dynamics and neural basis. Neuropsychologia 48: 3198–3204. Available: http://www.ncbi.nlm.nih.gov/entrez/query.fcgi?cmd=Retrieve&db=PubMed&list_uids=20600178&dopt=Abstract. Accessed 17 March 2014.

169. Zink CF, Pagnoni G, Martin-Skurski ME, Chappelow JC, Berns GS (2004) Human striatal responses to monetary reward depend on saliency. Neuron 42: 509–517. Available: http://www.ncbi.nlm.nih.gov/entrez/query.fcgi?cmd=Retrieve&db=PubMed&list_uids=15134646&dopt=Abstract.

DELAY DISCOUNT TASK META-ANALYSIS

1. Peters J, Büchel C (2010) Episodic future thinking reduces reward delay discounting through an enhancement of prefrontal-mediotemporal interactions. Neuron 66: 138–148. Available: http://www.ncbi.nlm.nih.gov/entrez/query.fcgi?cmd=Retrieve&db=PubMed&list_uids=20399735&dopt=Abstract. Accessed 10 March 2014.

2. Ballard K, Knutson B (2009) Dissociable neural representations of future reward magnitude and delay during temporal discounting. Neuroimage 45: 143–150. Available: http://www.ncbi.nlm.nih.gov/entrez/query.fcgi?cmd=Retrieve&db=PubMed&list_uids=19071223&dopt=Abstract.

3. Bickel WK, Pitcock JA, Yi R, Angtuaco EJC (2009) Congruence of BOLD response across intertemporal choice conditions: Fictive and real money gains and losses. J Neurosci 29:

8839–8846. Available: http://www.ncbi.nlm.nih.gov/entrez/query.fcgi?cmd=Retrieve&db=PubMed&list_uids=19587291&dopt=Abstract.

4. Hoffman WF, Schwartz DL, Huckans MS, McFarland BH, Meiri G, et al. (2008) Cortical activation druing delay discounting in abstinent methampetamine dependent individuals. Psychopharmacology (Berl) 201: 183–193. Available: http://www.ncbi.nlm.nih.gov/entrez/query.fcgi?cmd=Retrieve&db=PubMed&list_uids=18685833&dopt=Abstract.

5. McClure SM, Laibson DI, Loewenstein G, Cohen JD (2004) Separate neural systems value immediate and delayed monetary rewards. Science (80-) 306: 503–507. Available: http://www.ncbi.nlm.nih.gov/entrez/query.fcgi?cmd=Retrieve&db=PubMed&list_uids=15486304&dopt=Abstract.

6. Monterosso J, Ainslie G, Xu J, Cordova X, Domier CP, et al. (2007) Frontoparietal cortical activity of methamphetamine-dependent and comparison subjects performing a delay discounting task. Hum Brain Mapp 28: 383–393. Available: http://www.ncbi.nlm.nih.gov/entrez/query.fcgi?cmd=Retrieve&db=PubMed&list_uids=16944492&dopt=Abstract.

7. Peters J, Buchel C (2009) Overlapping and distinct neural systems code for subjective value during intertemporal and risky decision making. J Neurosci 29: 15727–15734. Available: http://www.ncbi.nlm.nih.gov/entrez/query.fcgi?cmd=Retrieve&db=PubMed&list_uids=20016088&dopt=Abstract.

8. Shamosh NA, Deyoung CG, Green AE, Reis DL, Johnson MR, et al. (2008) Individual differences in delay discounting. Psychol Sci 19: 904–911. Available: http://www.ncbi.nlm.nih.gov/entrez/query.fcgi?cmd=Retrieve&db=PubMed&list_uids=18947356&dopt=Abstract.

9. Volkow ND, Tomasi D, Wang GJ, Telang F, Fowler JS, et al. (2009) Hyperstimulation of striatal D2 receptors with sleep deprivation: Implications for cognitive impairment. Neuroimage 45: 1232–1240. Available: http://www.ncbi.nlm.nih.gov/entrez/query.fcgi?cmd=Retrieve&db=PubMed&list_uids=19349237&dopt=Abstract.

10. Wittmann M, Leland DS, Paulus MP (2007) Time and decision making: Differential contribution of the posterior insular cortex and the striatum during a delay discounting task. Exp Brain Res 179: 643–653. Available: http://www.ncbi.nlm.nih.gov/entrez/query.fcgi?cmd=Retrieve&db=PubMed&list_uids=17216152&dopt=Abstract.

11. Xu L, Liang ZY, Wang K, Li S, Jiang T (2009) Neural mechanism of intertemporal choice: From discounting future gains to future losses. Brain Res 1261: 65–74. Available: http://www.ncbi.nlm.nih.gov/entrez/query.fcgi?cmd=Retrieve&db=PubMed&list_uids=19185567&dopt=Abstract.

THEORY OF MIND TASK META-ANALYSIS

1. Blackwood N, Ffytche DH, Simmons A, Bentall R, Murray RM, et al. (2004) The cerebellum and decision making under uncertainty. Cogn Brain Res 20: 46–53. Available: http://www.ncbi.nlm.nih.gov/entrez/query.fcgi?cmd=Retrieve&db=PubMed&list_uids=15130588&dopt=Abstract.

2. Calarge C, Andreasen NC, O'Leary DS (2003) Visualizing how one brain understands another: A PET study of theory of mind. Am J Psychiatry 160: 1954–1964. Available: http://www.ncbi.nlm.nih.gov/entrez/query.fcgi?cmd=Retrieve&db=PubMed&list_uids=14594741&dopt=Abstract.

3. Decety J, Jackson PL, Sommerville JA, Chaminade T, Meltzoff AN (2004) The neural bases of cooperation and competition: An fMRI investigation. Neuroimage 23: 744–751. Available: http://www.ncbi.nlm.nih.gov/entrez/query.fcgi?cmd=Retrieve&db=PubMed&list_uids=15488424&dopt=Abstract.

4. Fossati P, Hevenor SJ, Graham SJ, Grady C, Keightley ML, et al. (2003) In search of the emotional self: An FMRI study using positive and negative emotional words. Am J Psychiatry 160: 1938–1945. Available: http://www.ncbi.nlm.nih.gov/entrez/query.fcgi?cmd=Retrieve&db=PubMed&list_uids=14594739&dopt=Abstract.

5. Gobbini MI, Koralek AC, Bryan RE, Montgomery KJ, Haxby J V (2007) Two takes on the social brain: A comparison of theory of mind tasks. J Cogn Neurosci 19: 1803–1814. Available: http://www.ncbi.nlm.nih.gov/entrez/query.fcgi?cmd=Retrieve&db=PubMed&list_uids=17958483&dopt=Abstract.

6. Hampton AN, Bossaerts P, O'Doherty JP (2008) Neural correlates of mentalizing-related computations during strategic interactions in humans. Proc Natl Acad Sci 105: 6741–6746. Available: http://www.ncbi.nlm.nih.gov/entrez/query.fcgi?cmd=Retrieve&db=PubMed&list_uids=18427116&dopt=Abstract.

7. Hill S, Kostelnik B, Holmes B, Goradia D, McDermott M, et al. (2007) fMRI BOLD response to the eyes task in offspring from multiplex alcohol dependence families. Alcohol Clin Exp Res 31: 2028–2035. Available: http://www.ncbi.nlm.nih.gov/entrez/query.fcgi?cmd=Retrieve&db=PubMed&list_uids=18034695&dopt=Abstract.

8. Kobayashi C, Glover GH, Temple E (2007) Children's and adult's neural bases of verbal and nonverbal "theory of mind." Neuropsychologia 45: 1522–1532. Available: http://www.ncbi.nlm.nih.gov/entrez/query.fcgi?cmd=Retrieve&db=PubMed&list_uids=17208260&dopt=Abstract.

9. Lissek S, Peters S, Fuchs N, Witthaus H, Nicolas V, et al. (2008) Cooperation and deception recruit different subsets of the theory-of-mind network. PLoS One 3: 2023. Available: http://www.ncbi.nlm.nih.gov/entrez/query.fcgi?cmd=Retrieve&db=PubMed&list_uids=18431500&dopt=Abstract.

10. Marjoram D, Job DE, Whalley HC, Gountouna VE, McIntosh AM, et al. (2006) A visual joke fMRI investigation into Theory of Mind and enhanced risk of schizophrenia. Neuroimage 31: 1850–1858. Available: http://www.ncbi.nlm.nih.gov/entrez/query.fcgi?cmd=Retrieve&db=PubMed&list_uids=16624578&dopt=Abstract.

11. Modinos G, Ormel J, Aleman A (2009) Activation of anterior insula during self-reflection. PLoS One 4: 4618. Available:

http://www.ncbi.nlm.nih.gov/entrez/query.fcgi?cmd=Retrieve&db=PubMed&list_uids=19242539&dopt=Abstract.

12. Moriguchi Y, Ohnishi T, Lane RD, Maeda M, Mori T, et al. (2006) Impaired self-awareness and theory of mind: An fMRI study of mentalizing in alexithymia. Neuroimage 32: 1472–1482. Available: http://www.ncbi.nlm.nih.gov/entrez/query.fcgi?cmd=Retrieve&db=PubMed&list_uids=16798016&dopt=Abstract.

13. Ochsner KN, Beer JS, Robertson ER, Cooper JC, Gabrieli JDE, et al. (2005) The neural correlates of direct and reflected self-knowledge. Neuroimage 28: 797–814. Available: http://www.ncbi.nlm.nih.gov/entrez/query.fcgi?cmd=Retrieve&db=PubMed&list_uids=16290016&dopt=Abstract.

14. Ochsner KN, Knierim K, Ludlow DH, Hanelin J, Ramachandran T, et al. (2004) Reflecting upon feelings: An fMRI study of neural systems supporting the attribution of emotion to self and other. J Cogn Neurosci 16: 1746–1772. Available: http://www.ncbi.nlm.nih.gov/entrez/query.fcgi?cmd=Retrieve&db=PubMed&list_uids=15701226&dopt=Abstract.

15. Perner J, Aichhorn M, Kronbichler M, Staffen W, Ladurner G (2006) Thinking of mental and other representations: The roles of left and right temporo-parietal junction. Soc Neurosci 1: 245–258. Available: http://www.ncbi.nlm.nih.gov/entrez/query.fcgi?cmd=Retrieve&db=PubMed&list_uids=18633791&dopt=Abstract.

16. Ruby P (2004) How would you feel versus how do you think she would feel? A neuroimaging study of perspective-taking with social emotions. J Cogn Neurosci 16: 988–999. Available: http://www.ncbi.nlm.nih.gov/entrez/query.fcgi?cmd=Retrieve&db=PubMed&list_uids=15298786&dopt=Abstract.

17. Ruby P, Decety J (2003) What you believe versus what you think they believe: A neuroimaging study of conceptual perspective-taking. Eur J Neurosci 17: 2475–2480. Available:

http://www.ncbi.nlm.nih.gov/entrez/query.fcgi?cmd=Retrieve&db=PubMed&list_uids=12814380&dopt=Abstract.

18. Schmitz TW, Kawahara TN, Johnson SC (2004) Metacognitive evaluation, self-relevance, and the right prefrontal cortex. Neuroimage 22: 941–947. Available: http://www.ncbi.nlm.nih.gov/entrez/query.fcgi?cmd=Retrieve&db=PubMed&list_uids=15193625 &dopt=Abstract.

19. Sebastian CL, Fontaine NMG, Bird G, Blakemore S-J, Brito SA De, et al. (2012) Neural processing associated with cognitive and affective Theory of Mind in adolescents and adults. Soc Cogn Affect Neurosci 7: 53–63. Available: http://www.ncbi.nlm.nih.gov/entrez/query.fcgi?cmd=Retrieve&db=PubMed&list_uids=21467048&dopt=Abstract. Accessed 21 February 2014.

20. Smoski M, Felder J, Bizzell J, Green SR, Ernst M, et al. (2009) fMRI of alterations in reward selection, anticipation, and feedback in major depressive disorder. J Affect Disord 118: 69–78. Available: http://www.ncbi.nlm.nih.gov/entrez/query.fcgi?cmd=Retrieve&db=PubMed&list_uids=19261334&dopt=Abstract.

21. Sommer M, Dohnel K, Sodian B, Meinhardt J, Thoermer C, et al. (2007) Neural correlates of true and false belief reasoning. Neuroimage 35: 1378–1384. Available: http://www.ncbi.nlm.nih.gov/entrez/query.fcgi?cmd=Retrieve&db=PubMed&list_uids=17376703&dopt=Abstract.

22. Sripada CS, Angstadt M, Banks S, Nathan PJ, Liberzon I, et al. (2009) Functional neuroimaging of mentalizing during the trust game in social anxiety disorder. Neuroreport 20: 984–989. Available: http://www.ncbi.nlm.nih.gov/entrez/query.fcgi?cmd=Retrieve&db=PubMed&list_uids=19521264&dopt=Abstract.

23. Uchiyama H, Seki A, Kageyama H, Saito DN, Koeda T, et al. (2006) Neural substrates of sarcasm: A functional magnetic-resonance imaging study. Brain Res 1124: 100–110. Available: http://www.ncbi.nlm.nih.gov/entrez/query.fcgi?cmd=Retrieve&db=PubMed&list_uids=17092490&dopt=Abstract.

24. Vanderwal T, Hunyadi E, Grupe DW, Connors CM, Schultz RT (2008) Self, mother and abstract other: An fMRI study of reflective social processing. Neuroimage 41: 1437–1446. Available: http://www.ncbi.nlm.nih.gov/entrez/query.fcgi?cmd=Retrieve&db=PubMed&list_uids=18486489&dopt=Abstract.

25. Vollm BA, Taylor ANW, Richardson P, Corcoran R, Stirling J, et al. (2006) Neuronal correlates of theory of mind and empathy: A functional magnetic resonance imaging study in a nonverbal task. Neuroimage 29: 90–98. Available: http://www.ncbi.nlm.nih.gov/entrez/query.fcgi?cmd=Retrieve&db=PubMed&list_uids=16122944&dopt=Abstract.

26. Wicker B, Ruby P, Royet JP, Fonlupt P (2003) A relation between rest and the self in the brain. Brain Res Rev 43: 224–230. Available: http://www.ncbi.nlm.nih.gov/entrez/query.fcgi?cmd=Retrieve&db=PubMed&list_uids=14572916&dopt=Abstract.
